# Large Language Models in Adverse Drug Reaction Detection and Pharmacovigilance: A Systematic Review of Current Applications, Challenges, and Future Directions

**DOI:** 10.3390/diagnostics16152435

**Published:** 2026-08-01

**Authors:** Tae You Kim, Won-Sik Oh, Dong-Hwa Jeong

**Affiliations:** 1 The Department of Healthcare and Artificial Intelligence, The Catholic University of Korea, Bucheon 14662, Republic of Korea; taeyoukim@catholic.ac.kr; 2 The Department of Artificial Intelligence, The Catholic University of Korea, Bucheon 14662, Republic of Korea; dodow@catholic.ac.kr

**Keywords:** large language models, pharmacovigilance, adverse drug reaction, adverse drug event, precision medicine, big data, drug safety, signal detection, clinical notes, literature screening

## Abstract

**Background/Objectives:** Pharmacovigilance workflows rely heavily on unstructured text across diverse sources. Here, we systematically reviewed how large language models (LLMs) are being explored as support tools for adverse drug reaction (ADR) detection, extraction, triage, and documentation, highlighting their potential for precision medicine and big data-enabled safety monitoring. **Methods:** Following the Preferred Reporting Items for Systematic Reviews and Meta-Analyses 2020 guidelines, we systematically searched PubMed, Scopus, and Web of Science for studies published between January 2022 and March 2026. Ultimately, 83 empirical studies satisfied the inclusion criteria. A narrative synthesis was conducted to address methodological heterogeneity across these studies. **Results:** LLM applications were concentrated in constrained information-extraction and classification tasks, including signal evaluation, clinical-note extraction, social media surveillance, and literature screening. Quantitative performance varied substantially by system design: error-correction prompting yielded an F1-score of 0.921 for ADR named entity recognition, whereas retrieval-augmented generation improved data-retrieval accuracy from 8.3% to 78.3%. Most studies were retrospective, benchmark-based, or proof-of-concept evaluations. Across 581 paired pre-consensus domain judgements, observed inter-rater agreement was 90.4% and Cohen’s κ was 0.837 (95% CI 0.772–0.895). Hallucination, low specificity, prompt sensitivity, narrow datasets, and weak external validation remained common limitations. **Conclusions:** Current evidence supports supervised, task-specific applications of LLMs for extraction, triage, retrieval, and documentation rather than autonomous pharmacovigilance decision-making. Prospective evaluation, external validation, transparent reporting, and accountable human oversight are required before high-stakes clinical or regulatory deployment.

## 1. Introduction

An adverse event (AE) is any untoward medical occurrence temporally associated with use of a medicinal product, without requiring a causal relationship. An adverse drug event (ADE) is medication-related harm and can include injury arising from appropriate use, medication error, or other medication-use processes. An adverse drug reaction (ADR) is a noxious and unintended response for which a causal relationship to the medicinal product is at least reasonably suspected. These distinctions are important because pharmacovigilance systems must identify broad safety events while supporting the narrower task of causal assessment. The clinical burden is substantial: the World Health Organization reports that medication-related harm accounts for a major share of preventable harm in health care and that medication errors alone cost an estimated USD 42 billion globally each year [1]. Clinically actionable signals are often embedded in unstructured clinical notes, discharge summaries, spontaneous-report narratives, biomedical literature, and patient-generated content, making timely surveillance labor-intensive and difficult to scale [2,3,4].

Traditional natural language processing (NLP) and machine learning (ML) approaches have long been investigated to address this bottleneck. Rule-based methods, classical ML pipelines, and later transformer-based architectures, such as bidirectional encoder representation (BERT), BioBERT, and other domain-specific variants, have improved the classification of adverse-event reports and the mining of drug-safety information [5,6,7,8]. Nevertheless, these approaches often lack the contextual nuance required for precision safety monitoring, as they require task-specific engineering, substantial labeled data, or tightly constrained problem definitions. Consequently, many pharmacovigilance workflows still rely heavily on manual screening and expert interpretation to establish definitive clinical assessments of ADRs [2,5].

The rapid emergence of large language models (LLMs) has introduced a new paradigm for biomedical text analysis and safety surveillance. General-purpose and domain-adapted LLMs, including GPT-family models, Gemini, Claude, Llama, and clinical language models, are being evaluated for detecting drug–drug interactions (DDIs), extracting ADE-related phenotypes from clinical notes, and supporting precision pharmacovigilance [4,9,10,11,12]. This development also reflects the broader evolution of clinical AI toward predictive analytics, real-time monitoring, and medication-safety support, while preserving the need for privacy, bias control, and human oversight [13].

However, the enthusiasm for deploying LLMs as surveillance tools in medication safety must be balanced by rigorous clinical scrutiny. Existing studies indicate that although LLMs perform well in selected extraction tasks, critical operational limitations persist. These include low specificity (often leading to false-positive risk overcalling), hallucinated or nonfactual outputs, inconsistent clinical reasoning, prompt sensitivity, and a lack of prospective clinical validation. In precision medicine and pharmacovigilance, these limitations transcend mere technical inconveniences, representing major barriers to safe, credible, and clinically accountable deployment [2,4,11,14].

While several recent review papers have explored generative artificial intelligence (GAI) in medication safety [2,7,14], they predominantly take the form of broad scoping reviews or narrative discussions centered on theoretical workflow integrations. These previous works provide valuable conceptual overviews but generally lack granular, task-specific performance syntheses and structured risk-of-bias assessments.

To address this gap, this review systematically synthesizes 83 empirical studies, compares performance across constrained extraction, retrieval, workflow, and open-ended reasoning tasks, and evaluates methodological quality using an evidence-limitation framework adapted for heterogeneous LLM studies. It further distinguishes isolated model evaluations from broader human-in-the-loop workflow interventions, maps recurring failure modes to practical governance controls, and identifies priorities for future consensus-based reporting standards.

Accordingly, this systematic review is aimed at synthesizing the emerging literature on the utilization of LLMs in ADR detection, ADE phenotyping, and precision pharmacovigilance. Specifically, it addresses the following questions: (1) which extraction and evaluation tasks are currently being studied, (2) which real-world data sources are being leveraged, (3) which LLMs and model configurations are being applied, (4) how these systems are being evaluated for extraction accuracy and utility, and (5) which methodological, clinical, and implementation barriers are being most consistently reported.

## 2. Materials and Methods

### 2.1. Study Design

This study was designed as a systematic review of the emerging literature on the utilization of LLMs in ADR, ADE, and pharmacovigilance-related research. The primary objective was to identify, categorize, and synthesize how LLMs are being applied across drug-safety-relevant tasks, including adverse-event extraction, DDI-related evaluation, literature screening, safety-document retrieval, structured-case processing, and expert-support workflows. A secondary objective was to clarify where the current evidence appears strongest, where it remains methodologically weak, and which application areas appear most plausible for near-term implementation.

Because the literature is evolving rapidly, spanning empirical studies and broader contextual material, this review was designed to simultaneously summarize the empirical evidence and apply interpretive literature for framing. This distinction is crucial because the pharmacovigilance literature on LLMs remains fragmented across data sources, tasks, and publication types.

This systematic review was conducted and reported in accordance with the Preferred Reporting Items for Systematic Reviews and Meta-Analyses (PRISMA) 2020 guidelines [15]. The review protocol was prospectively registered on the Open Science Framework Registries before data extraction (Project ID: B8u9e; registration embargoed until 30 September 2026).

### 2.2. Review Questions

The review was guided by the following questions:

- Beyond basic adoption, how are LLMs fundamentally shifting or augmenting traditional workflows in ADR, ADE, and pharmacovigilance research?
- In which specific functional tasks (e.g., extraction, signal evaluation, literature screening, and expert-support workflows) do LLMs demonstrate a distinct comparative advantage or disadvantage against traditional NLP models?
- How do current studies navigate the complexities of diverse data sources (e.g., clinical notes, spontaneous reports, and social media), particularly regarding data privacy and the handling of unstructured, noisy text?
- What combinations of LLM architectures, prompting strategies (e.g., zero-shot, few-shot, or retrieval-augmented generation [RAG]), and model configurations yield the most robust outcomes for pharmacovigilance tasks?
- What is the translational gap between reported performance metrics in controlled experimental settings and their practical utility or readiness for real-world clinical integration?
- How do existing studies address critical operational barriers, including hallucination, low specificity, and prompt sensitivity, and what frameworks are proposed to meet the strict regulatory standards required in pharmacovigilance?

### 2.3. Information Sources and Search Strategy

A structured literature search was conducted across three databases: PubMed, Scopus, and the Web of Science (WoS), which were selected to balance biomedical relevance, interdisciplinary coverage, and citation breadth. The search logic combined two conceptual domains connected by the Boolean AND operator: (1) LLMs and generative language systems and (2) ADR-, ADE-, and pharmacovigilance-related concepts.

The LLM-domain search terms included “large language model,” “large language models,” “LLM,” “GPT,” “ChatGPT,” “Gemini,” “Claude,” and “Llama.” The pharmacovigilance and drug-safety terms included “adverse drug reaction,” “adverse drug reactions,” “adverse drug event,” “adverse drug events,” “ADR,” “ADE,” “pharmacovigilance,” “drug safety,” and “medication safety.” Database-specific syntax was adapted to title, abstract, and keyword fields as required. The complete database-specific Boolean strings, limits, coverage period, and final search date are provided in Supplementary Materials, Table S9.

Search outputs from the databases were exported, merged, and deduplicated before screening. A final post-registration search update was subsequently conducted on 30 March 2026, covering literature published between 1 January 2022 and 30 March 2026. Before cross-database deduplication, the final searches identified 662 records in total (107, 404, and 151 from PubMed, Scopus, and WoS, respectively).

### 2.4. Eligibility Criteria

The inclusion and exclusion criteria applied in this review are summarized in Table 1.

Because the recent surge of LLM literature is concentrated primarily in the post-2022 period, studies from 2022 onward constituted the core evidence era. Earlier transformer- or BERT-based studies were selectively retained as background for interpretation when necessary; however, they were not included in the final empirical set.

### 2.5. Study Selection and Layered Inclusion Strategy

After cross-database deduplication, two reviewers (T.Y.K. and W.-S.O.) applied the eligibility criteria to titles/abstracts and then to full-text reports using a structured consensus process. Both reviewers examined the candidate records and discussed uncertain or discordant cases until a joint decision was reached. Independent pre-consensus inclusion/exclusion decisions were not retained; therefore, a valid screening-stage Cohen’s κ could not be calculated retrospectively. Contextual reviews and perspective articles informed framing and interpretation but were not included among the 83 empirical studies in the systematic synthesis.

### 2.6. Data Extraction

Data were extracted into a structured review matrix. For each study, the extracted variables included title, year, study type, task, data source, dataset or corpus, sample or corpus size when reported, model or LLM, comparator or baseline, evaluation metrics, performance estimates, measures of uncertainty when available, principal findings, key limitations, and relevance to pharmacovigilance. Missing or unavailable information was recorded as not reported rather than inferred. The complete study-level matrix is provided in Supplementary Materials, Table S1.

### 2.7. Synthesis Strategy

Given the methodological heterogeneity of the literature, quantitative pooling was not considered appropriate. Instead, a narrative synthesis approach was adopted, proceeding across two levels. First, the empirical core studies were analyzed in terms of task subtype, data source, model configuration, and recurrent performance patterns. Second, the contextual narrative-support literature was used to interpret broader themes, including the maturity of the field, implementation barriers, and the likely near-term role of LLMs in pharmacovigilance tasks.

### 2.8. Evaluation Dimensions and Evidence-Limitation Assessment

Conventional risk-of-bias tools designed for randomized interventions could not be applied uniformly to the 83-study empirical core, which comprised methodological studies, retrospective evaluations, benchmark experiments, and workflow pilots. Review and contextual papers were used only to support interpretation and were not included in the RoB assessment. We therefore evaluated recurring evidence limitations, including dataset representativeness, task reproducibility, model transparency, Evaluation Design, comparator adequacy, Outcome Reporting, and External Validity.

The assessment framework was adapted from PROBAST+AI, TRIPOD+AI, TRIPOD-LLM, CLAIM, CONSORT-AI, and DECIDE-AI [16,17,18,19,20,21]. It comprised 15 signaling questions across seven domains: Data Quality, Task Definition, Model Transparency, Evaluation Design, Comparator Strength, Outcome Reporting, and External Validity. The framework was used as an evidence-limitation appraisal rather than represented as a prospectively validated universal RoB instrument for LLM studies. Domain definitions, signaling questions, source-framework mappings, and adaptation rationales are provided in Supplementary Materials, Table S6.

Two reviewers (T.Y.K. and W.-S.O.) independently assessed all 83 studies before any discussion of ratings. A domain was classified as low-risk when all signaling questions were answered with “Yes,” high-risk when any question was answered with “No,” and unclear otherwise. Because the categories were nominal, reliability was summarized using unweighted Cohen’s κ and observed agreement. Overall estimates pooled paired judgements across questions or domains; 95% confidence intervals were estimated with 10,000 percentile cluster-bootstrap resamples at the study level. Paired pre-consensus ratings and reliability estimates are reported in Supplementary Tables S7, S8 and S10.

### 2.9. Use of Generative Artificial Intelligence

Claude (Sonnet 4.6, Anthropic PBC, San Francisco, CA, USA) was used during manuscript preparation and revision in 2026 for text drafting, structural editing, language refinement, and formatting support for the study-level extraction matrix. Inputs were limited to bibliographic metadata, published article text, and study-level extracted information; no identifiable patient-level or confidential clinical data were supplied. Model outputs were treated as drafts and checked by the authors against the source articles and extraction matrix. The model did not make eligibility decisions, assign RoB ratings, perform statistical calculations, derive the appraisal framework, or determine the interpretation and conclusions. The authors retained full responsibility for all analytic and editorial decisions. Exact session dates and a complete prompt/output archive were not prospectively retained.

## 3. Results

### 3.1. Overview of the Included Literature

**Figure 1:**
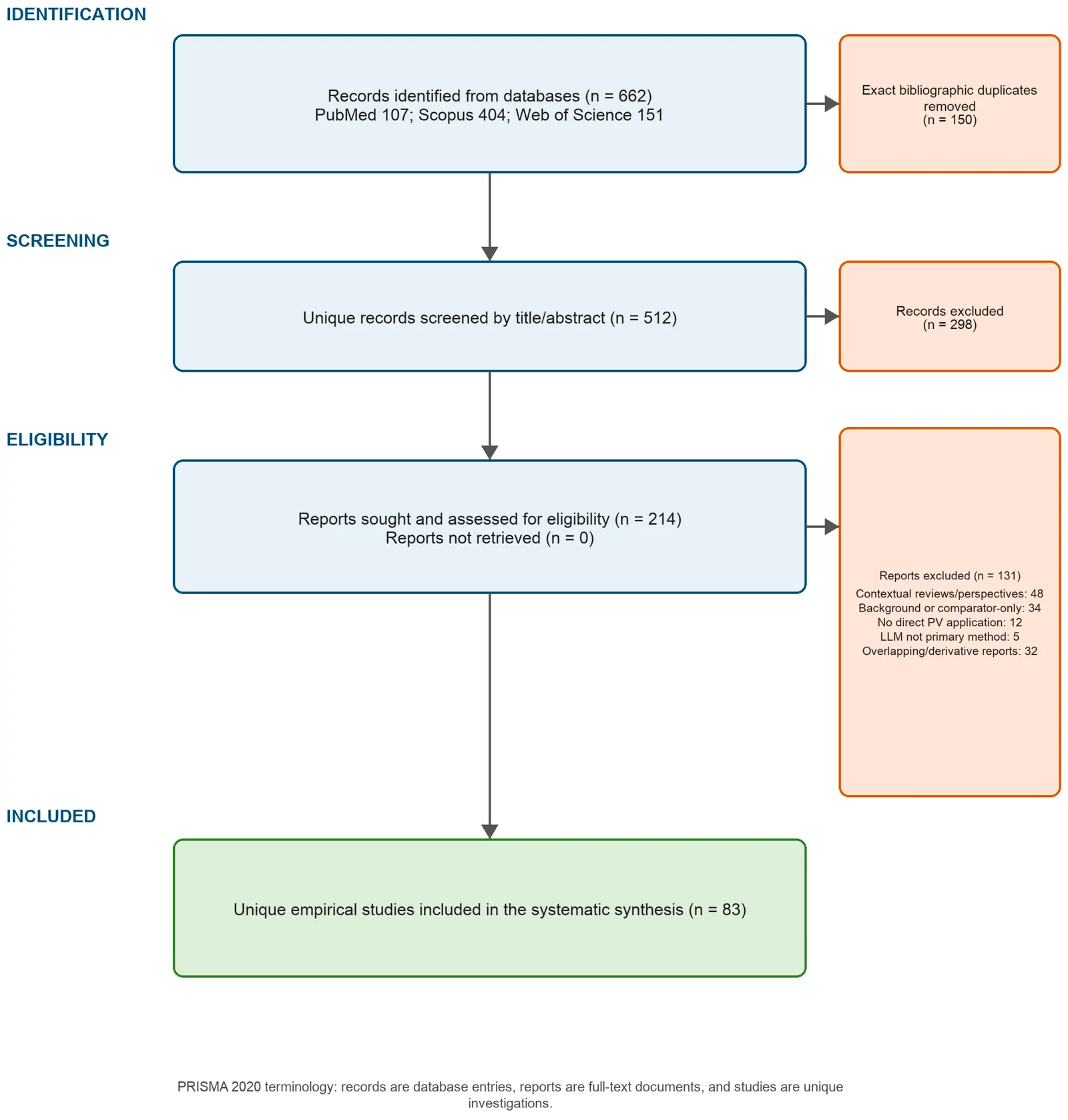
PRISMA 2020 flow diagram distinguishing database records, full-text reports, and unique studies. Searches identified 662 records (PubMed 107, Scopus 404, and Web of Science 151); 150 exact bibliographic duplicates were removed and 512 unique records were screened. After 298 title/abstract exclusions, 214 reports were assessed in full, 131 were excluded for the itemized reasons shown, and 83 unique empirical studies were included.

**Figure 2:**
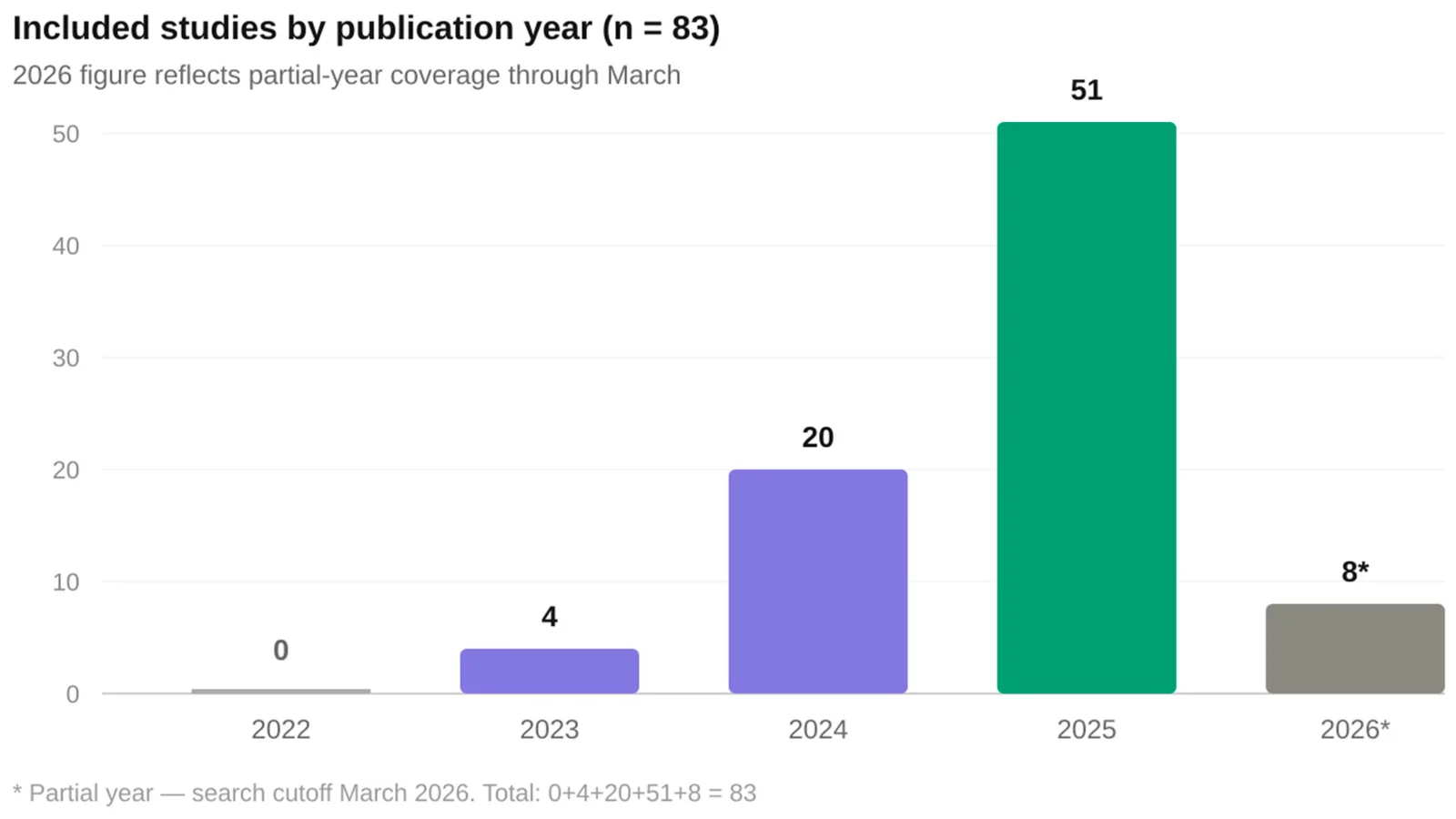
Number of included studies by publication year (2022–2026). The marked increase from 2024 onward reflects the accelerating adoption of LLMs in pharmacovigilance research; the 2026 figure reflects partial-year coverage through the March search cutoff.

The searches identified 662 database records, of which 150 exact bibliographic duplicates were removed before title/abstract screening. Of 512 unique records screened, 298 were excluded and 214 full-text reports were assessed; no sought reports were unavailable. A further 131 reports were excluded, leaving 83 unique empirical studies. The 32 full-text exclusions classified as overlapping were not duplicate database records: they were distinct publications describing the same underlying dataset, cohort, or derivative analysis as another report, without a separable empirical contribution relevant to this review (Figure 1 and Figure 2). Full exclusion categories and counts are reported in Supplementary Materials, Table S3.

**Figure 3:**
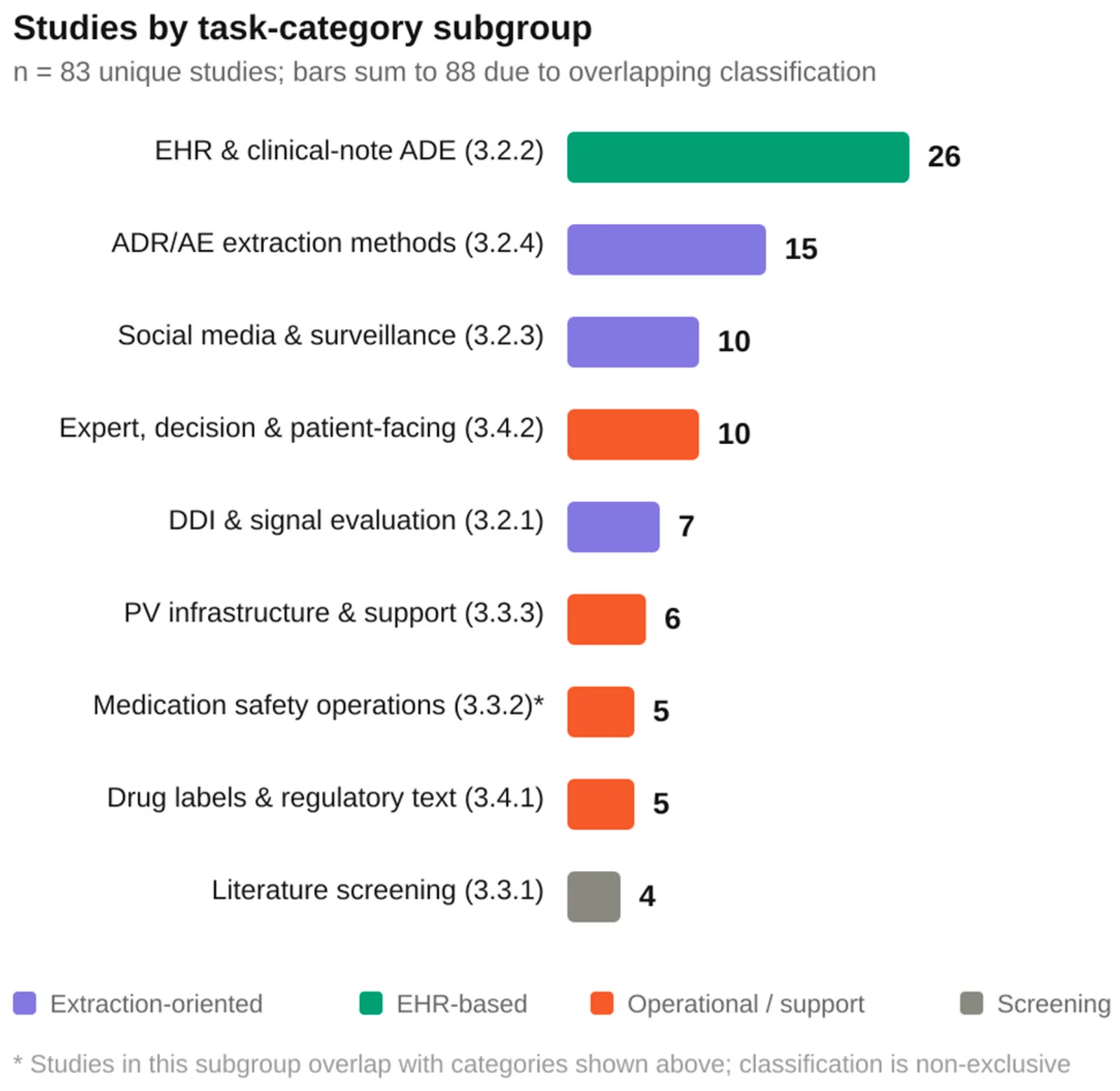
Distribution of the 83 included studies across nine task-category subgroups, corresponding to the subsections presented in Section 3.2, Section 3.3 and Section 3.4.

A comprehensive study-level comparison is provided in Supplementary Materials, Table S1, including data source and dataset, LLM or model, pharmacovigilance task, evaluation metrics, reported sample or corpus size, performance, uncertainty measures, key findings, and appraisal-based limitations. The 83 studies span direct signal detection and extraction, operational pharmacovigilance workflows, and document- or user-facing medication-safety support. Some studies contributed to more than one functional category; therefore, category counts exceed the number of unique studies. The functional subgroup distribution is summarized in Figure 3.

Across these layers, LLMs generally performed more consistently in constrained information-extraction and classification tasks than in open-ended clinical or pharmacological reasoning. Table 2 presents representative quantitative results, while Supplementary Table S1 provides the comprehensive 83-study comparison requested for cross-study interpretation.

### 3.2. Direct Signal Detection and Extraction Tasks

#### 3.2.1. DDI and Signal-Evaluation Studies

A notable empirical subtype identified in this review encompassed DDI- and signal-evaluation tasks (*n* = 7). These studies examined whether LLMs could identify clinically relevant DDIs, detect safety-relevant signals, or facilitate pharmacovigilance case-level assessments [9,22,23,24,25]. This subtype is particularly crucial because it sits close to real pharmacovigilance decision-making, providing some of the most clinically interpretable evidence in the current literature. LLMs evaluated in this subgroup included GPT-family models, Gemini, Claude, and task-specific chain-of-thought enhanced language (CoTEL) applied to DDI triplet extraction (CoTEL-D3X) and interaction-classification tasks.

Across DDI and signal-evaluation studies, high sensitivity was frequently accompanied by low specificity and thus a high false-positive rate. Sicard et al. [9] found that ChatGPT, Gemini, and Claude frequently identified interactions absent from the reference standard. This pattern can increase downstream review burden even when recall-oriented metrics appear favorable. Knowledge grounding and structured contextual reasoning improved reliability in other evaluations [26], indicating that system design may matter as much as base-model choice.

The DDI subtype also illustrates the criticality of the prompt design and reasoning scaffold in determining output quality. CoTEL architectures, such as the CoTEL-D3X [24], consistently outperformed naive prompting configurations on structured extraction benchmarks. These findings support a design principle that is increasingly visible across the broader LLM literature: structured reasoning and RAG are not optional enhancements; rather, they are functional prerequisites for adequate performance in pharmacovigilance-adjacent tasks. The clinical implication is that DDI-related LLM tools cannot be evaluated or deployed using base-model benchmarks alone; system-level design, knowledge integration, and specificity-oriented evaluation must be central to any deployment framework.

#### 3.2.2. Electronic Health Record and Clinical-Note Adverse Drug Event Detection

The largest empirical subtype focuses on clinical notes and other EHR-derived narratives (*n* = 26). These studies examined the utilization of LLMs to identify ADEs, extract drug–event relations, or classify causally relevant drug–AE pairs embedded in discharge summaries or other unstructured clinical text [27,28,29,30]. This subtype is technically relevant because a substantial amount of clinically crucial medication-safety information is documented only in narrative form and may not be represented in structured data fields. Ensuring that this information is systematically captured has long been a central challenge in hospital pharmacovigilance, with LLMs representing a potentially scalable approach for addressing it.

Representative studies demonstrate the range of what has been achieved. For instance, Zhang et al. [31] reported that applying error-correction prompt engineering with GPT-4 yielded an F1-score of 0.921 on ADR named entity recognition (NER) tasks, outperforming fine-tuned LLM Meta AI (Llama) and DeepSeek baselines on the same corpora. Li et al. [32] demonstrated that ensembles integrating deep learning and fine-tuned LLMs significantly improved NER performance across vaccine adverse-event reporting system (VAERS) and social media sources, with cross-source generalization representing a key practical advantage over single-model approaches. Koon et al. [28] observed that generative LLMs could meaningfully predict ADR relations in discharge summaries, whereas Prioleau et al. [29] demonstrated that token- and span-based NER approaches yielded different performance profiles depending on the annotation scheme and text source.

Compared with broader expert-facing reasoning tasks, this subtype appears to be among the most technically promising LLM application areas. Studies in this group consistently suggest that LLMs improve the extraction of medication-related AE information, potentially outperforming or complementing existing ML baselines in constrained extraction contexts. Concurrently, persistent limitations include dependence on documentation quality, variation in note structure across institutions and languages, and residual performance gaps in causal-relation identification. Moreover, performance is consistently stronger for entity detection than for causality attribution, a distinction that matters greatly in clinical pharmacovigilance, where causality is the central legal and safety question. Fine-tuning domain-specific corpora generally improves performance, although it introduces generalizability issues when training narrow or institution-specific corpora. For instance, Cheligeer et al. [33] developed a three-module LLM-based framework using open-source models (Llama3, Mistral-7B, Gemma, and Phi-3). The model was used to detect pulmonary embolism as a hospital-acquired AE from unstructured electronic medical record (EMR) narratives, achieving sensitivity and specificity of 87.5–100% and 94.9–98.9%, respectively, across evaluation conditions.

#### 3.2.3. Social Media and Surveillance-Oriented Extraction

Another distinct subset of empirical studies focuses explicitly on extracting adverse events and monitoring safety signals from patient-generated content and social media platforms (*n* = 10).

These studies sourced data from X (formerly Twitter), Reddit, and VAERS-associated text to identify AE mentions, extract entities, classify ADR-related content, or study user-reported safety signals in noisier, less structured environments [32,34,35,36,37]. This line of work broadens pharmacovigilance beyond traditional spontaneous reporting systems, reflecting an increasing interest in patient-generated safety signals as a complement to institutional and regulatory data streams. The rationale is compelling: patients and caregivers often describe medication experiences in public digital spaces before, or instead of, formally reporting to pharmacovigilance centers. These signals may capture real-world adverse experiences that institutional systems traditionally undercount.

The reviewed studies indicate that LLMs contribute meaningfully in this field, particularly when integrated with fine-tuning, ensemble methods, or carefully designed extraction pipelines. Yazdani et al. [35] demonstrated that LLM-generated synthetic tweet data could augment NER performance for ADE detection, addressing the chronic data scarcity associated with social media pharmacovigilance. Daluwatte et al. [36] used a fine-tuned PubMedBERT model to identify COVID-19 vaccine AEs from web and social media content, demonstrating the feasibility of event-specific surveillance.

However, social media surveillance introduces distinct challenges that differentiate it from clinical-text extraction. These include highly informal and ambiguous language, slang and drug synonyms, domain shift between training and deployment corpora, and highly variable reliability of user-generated content. A model trained on one social media platform or vaccine campaign may not transfer reliably to a different therapeutic context. Carpenter et al.’s [38] research using GPT-3 to generate drug-of-abuse synonym lexicons illustrates one creative approach to addressing the informal language challenge; however, the broader reproducibility and transferability of such methods across drug classes and platforms have not been demonstrated. Overall, social media surveillance may be a viable yet technically demanding application area that benefits substantially from targeted fine-tuning and domain-specific pipeline designs.

#### 3.2.4. ADR/AE Extraction Methods as a Distinct Methodological Subgroup

A smaller yet methodologically relevant subset of empirical studies focuses more explicitly on method development for ADR/AE extraction, NER, and relation extraction (*n* = 15) [31,39,40,41,42,43,44]. These studies offered systematic insight into how prompt designs, error-correction strategies, fine-tuning, and hybrid pipelines influence ADR extraction performance, providing a more granular evidence base for understanding why certain extraction approaches outperform others. Unlike the clinical outcome-focused studies in Section 3.2.2, this subgroup treats the extraction method itself as the primary object of investigation.

Several consistent findings emerge across this subgroup. First, the zero-shot prompting of large general-purpose models, while capable of producing plausible-looking extractions, typically underperforms fine-tuned task-specific systems on established benchmarks. Second, error-correction prompting, in which the model is explicitly prompted to identify and correct its own extraction errors, substantially bridges this gap, as demonstrated by Zhang et al. [31], with an F1-score of 0.921 on ADR NER. Third, the inline-annotation approach (where the model annotates entities within the text) tends to outperform entity-only extraction approaches in clinical text settings, although the advantage varies across corpora [40]. Fourth, the integration of a dynamic knowledge-aware component with LLM-based NER, as explored by Qiu et al. [37], improves performance on rare and ambiguous ADR entity types that simpler models consistently miss. Collectively, this methodological subgroup provides the mechanistic foundation for the performance patterns observed more broadly in Section 3.2.1, Section 3.2.2 and Section 3.2.3.

### 3.3. Operational Pharmacovigilance Workflows

#### 3.3.1. Literature Screening and Retrieval Support

A smaller but conceptually relevant study subgroup addresses literature screening, documentation retrieval, or workflow-oriented search support for pharmacovigilance (*n* = 4 core empirical studies, with one methodological-framework paper included for a comparative scope) [10,45,46,47,48]. These studies explored whether LLMs could identify relevant articles, retrieve safety documentation, or translate natural language queries (NLQ) into structured retrieval actions. The current evidence suggests that LLMs may be particularly key to sensitivity-oriented screening or retrieval tasks, especially when structured prompting, examples, or business-context documents are added. Li et al. [10] found that LLMs demonstrated robust performance in facilitating literature screening for drug safety, with performance varying substantially with the model type and prompting strategy. Painter et al. [45] reported that GPT-4 augmentation with a business-context document improved NLQ-to-structured query language (SQL) generation accuracy from 8.3% to 78.3% for pharmacovigilance data-retrieval queries. This result illustrates the potential and prompt sensitivity of retrieval-augmented LLM systems.

As a methodological framework contribution rather than a direct empirical evaluation, Ma et al. [49] proposed an explainable LLM framework for classifying drug-induced liver injury literature, demonstrating the feasibility of automated article classification within a high-specificity domain. Collectively, these results suggest that LLMs may be valuable first-pass screening tools when sensitivity maximization is the goal, with human experts providing the final filter for specificity. The principal limitation is that LLM performance remains highly sensitive to prompt quality, retrieval-context specificity, and the structural consistency of the target literature. Consequently, deploying these models in formal systematic reviews or regulatory evidence-screening contexts would require substantially more extensive validation than is currently available in the literature.

#### 3.3.2. Medication Safety Operations

This operational subgroup comprises medication-safety tasks, such as structured medication extraction, dispensing-error detection, and related operational safety functions (*n* = 5) [50,51,52,53,54].

This subgroup is notable as it offers a practical route toward near-term implementation: targeted, bounded safety operations, such as labeling-error detection, polypharmacy flagging, or direction-error screening, may represent more tractable deployment contexts than fully autonomous pharmacovigilance reasoning. The key insight is that these tasks are operationally constrained, verifiable by structured reference standards, and directly connected to patient-harm mitigation. LLMs utilized in this context function primarily as structured classifiers rather than general reasoning systems, an operational role that aligns well with their current technical strengths.

#### 3.3.3. Pharmacovigilance Infrastructure and Support Systems

A related subgroup encompasses studies on documentation support, guardrails, case-documentation assistance, and broader infrastructure-level support for pharmacovigilance or medical safety (*n* = 6) [55,56,57,58,59,60]. These studies often explore how LLMs can be embedded within safer workflows rather than how they perform on a single benchmark task. As a contextual framework contribution, Hakim et al. [61] provided a position-paper analysis of the criticality of integrating guardrails with LLMs in pharmacovigilance and other safety-critical settings, arguing that unrestricted LLM outputs in drug-safety workflows pose unacceptable risks, while structured output control, factual grounding, and human-in-the-loop design are prerequisites for responsible deployment. Evaluating a ChatGPT-based tool for case-documentation assistance in an industry pharmacovigilance department, Benaïche et al. [56] reported significant time conservation in drafting case-documentation letters without requiring extensive user training, indicating practical documentation-level workflow value. Painter et al. [45] demonstrated infrastructure-level value through the NLQ-to-SQL pipeline, where LLM integration enabled nonspecialist staff to query safety databases more effectively.

### 3.4. Document-Facing and User-Facing Medication Safety Support

#### 3.4.1. Drug Label and Regulatory Text Processing

A smaller but highly relevant niche in the literature involves the deployment of LLMs to process drug labels, structured product labels, medication guides, and other regulatory safety documents (*n* = 5) [48,60,62,63,64]. Although these documents are highly structured by regulatory convention, they remain voluminous, technically dense, and challenging for nonspecialist users to navigate. Studies in this area suggest that generative models can match or exceed previous methods in extracting structured drug-safety information from prescription labels under constrained conditions. Gisladottir et al. [62], working with Food and Drug Administration (FDA) prescription drug labels, confirmed that LLMs effectively extracted structured safety information from complex label text, although performance depended substantially on consistency of the label format. Wu et al. [63] employed the AskFDALabel system to demonstrate enhanced AE annotation, profiling, and classification from FDA-labeled documents, highlighting the implications for regulatory review efficiency and downstream signal detection. Ying et al. [48] explored the text summarization of drug-labeling documents using ChatGPT, confirming that summaries were readable and informative but required careful quality control to prevent the omission of safety-critical content.

#### 3.4.2. Expert Support, Decision Support, and Patient-Facing Applications

A further subgroup comprises expert-facing support, decision support, and patient-facing drug-information studies, encompassing direct comparisons between chatbot outputs and pharmacovigilance specialists or clinicians, as well as user-facing medication-safety applications (*n* = 10) [2,11,44,55,61,65,66,67,68,69]. The current evidence suggests that although LLMs may provide useful initial structuring, information retrieval, or triage support, their outputs remain highly inconsistent for autonomous deployment in high-stakes pharmacovigilance decision-making.

Huang et al. [65] compared ChatGPT against clinical pharmacists across a range of clinical pharmacy tasks, including ADR recognition; they observed that while ChatGPT performed well on drug counseling queries, it was substantially weaker than pharmacists on complex-ADR recognition tasks. Montastruc et al. [11] raised the question of whether AI chatbots could replace clinical pharmacologists and concluded that current LLMs lack the domain-specific reasoning reliability and regulatory accountability required for autonomous pharmacovigilance roles despite their impressive conversational fluency. Pandya et al. [55] presented a causality-assessment case using ChatGPT for toxic epidermal necrolysis, revealing that ChatGPT produced ambiguous results compared with World Health Organization–Uppsala Monitoring Center (WHO–UMC) and Naranjo-scale assessments, emphasizing the need for caution in any causality-critical application.

A related set of studies examined patient- and user-facing drug-information tasks. Pariente et al. [70] evaluated the ability of ChatGPT to answer drug-safety questions, concluding that outputs were generally readable and often directionally correct, although they contained factual gaps that would be clinically relevant within a patient-facing context. Sheikh et al. [71] evaluated ChatGPT’s assessment of nonprescription medication safety in patients with kidney disease, finding that while its output accuracy was acceptable in routine cases, it was insufficient for edge cases and complex polypharmacy scenarios. Consequently, the central concern within this subgroup is not readability alone; it expands to reliability. If generated content is easier to understand but incomplete, ambiguous, or clinically misleading, the apparent usability benefit may directly compromise patient safety.

Dezoteux et al. [69] reported that a privacy-preserving LLM could automate detection of in-hospital drug-hypersensitivity reactions, illustrating an isolated task-specific evaluation. By contrast, Ong et al. [2] evaluated an LLM embedded within a broader clinical decision-support and human workflow across 16 specialties. Because the intervention combined the model with workflow redesign and human review, its outcome improvement cannot be attributed solely to the LLM’s intrinsic capability. We therefore distinguish isolated model evaluations from socio-technical workflow interventions and use the latter to inform implementation feasibility, not claims of standalone model reliability.

### 3.5. Cross-Cutting Evaluation Patterns

Across empirical subgroups, conventional predictive metrics—sensitivity, specificity, accuracy, F1-score, and AUROC—were used when a reference standard was available, whereas reasoning, decision-support, and documentation studies often relied on expert acceptability or workflow-efficiency outcomes. Measures of uncertainty were inconsistently reported; many point estimates lacked confidence intervals, dispersion estimates, or tests of comparative performance. Consequently, isolated point estimates were treated descriptively rather than as evidence of statistically established superiority.

Second, studies utilizing prompting strategies, reasoning scaffolds, retrieval augmentation, or hybrid model designs consistently yielded better performance compared with those relying exclusively on naive or zero-shot prompting [25,41,72]. This pattern is not incidental; it reflects the fundamental architecture of current LLMs, which are powerful pattern-matching and text-generation systems that perform best when provided with structured contextual constraints. The implication for research and deployment is that LLM performance metrics obtained from naive prompting configurations are likely to simultaneously underestimate achievable performance and underestimate the system-design complexity required to reliably approach that performance in practice.

Third, the strongest apparent performance is consistently observed in narrowly defined, benchmark-like tasks with well-annotated reference data, rather than in open-ended or prospectively evaluated operational settings. This creates a systematic optimism bias in the current evidence base that researchers and practitioners must account for when interpreting reported results.

### 3.6. Cross-Cutting Limitations

Despite the breadth of emerging applications, several limitations recur across the literature with sufficient frequency to be considered structural characteristics of the current evidence base. These recurring limitations are summarized in Table 3.

Low specificity and false-positive overcalling account for the most consistently reported performance limitations, particularly in DDI-evaluation and signal-detection tasks [8,53,65]. Hallucinated or nonfactual outputs, including invented drug names, fabricated interaction mechanisms, and unsupported causality claims, have been reported across multiple subtypes, representing a safety-critical concern that differs qualitatively from ordinary prediction errors. Unlike a false positive that is simply incorrect, a hallucinated output may be internally coherent and clinically plausible in form while being factually unfounded, further complicating detection for nonspecialist reviewers [61,73].

Prompt sensitivity is a second major structural limitation: performance on identical tasks may vary substantially with minor changes in prompt phrasing, instruction ordering, or system-prompt configuration. This variability has been documented across NER, DDI detection, literature-screening, and question-answering tasks, substantially complicating the interpretation of reported benchmark results [48,64].

Narrow or highly task-specific training datasets represent a third limitation: the majority of included studies evaluate models on small, retrospective, or highly controlled corpora that may not reflect the linguistic and clinical heterogeneity encountered in real-world pharmacovigilance practice [73].

Limited external validation represents the fourth recurrent structural limitation: only a few studies replicate findings across multiple institutions, languages, or data sources, with prospective real-world implementation studies remaining scarce [8].

Finally, reproducibility was limited by absent prompt documentation, unreported generation settings, single-run evaluations without variance estimates, unstated model versions, and inadequate reporting of computational and deployment requirements. Together with limited external validation and workflow confounding, these gaps restrict independent replication and transportability.

### 3.7. Risk-of-Bias Summary and Inter-Rater Reliability

**Figure 4:**
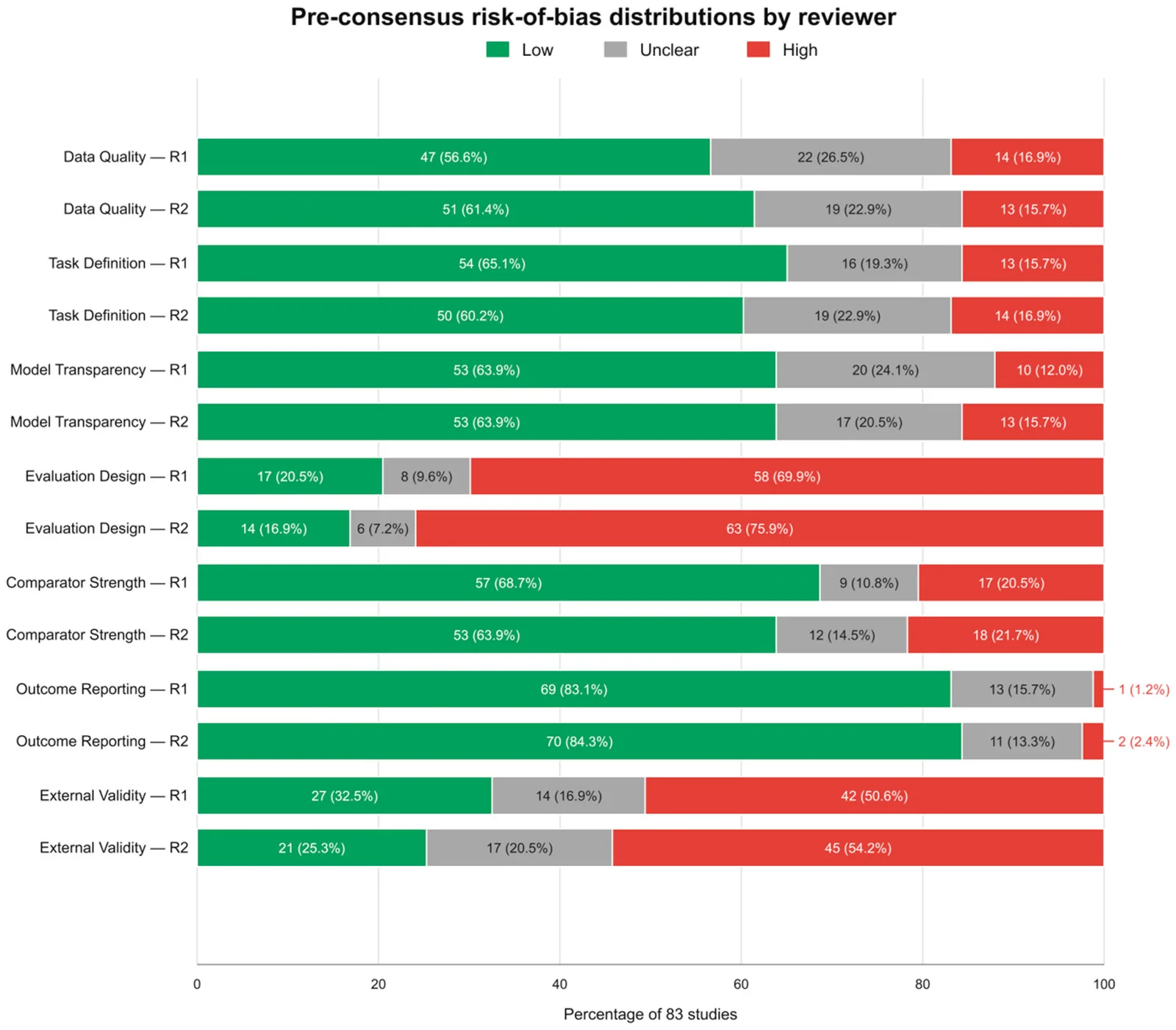
Pre-consensus domain-level risk-of-bias distributions for each reviewer across the 83 included studies. Percentages use 83 studies as the denominator for every bar. R1 = Rater 1; R2 = Rater 2.

The two reviewers’ pre-consensus domain distributions were broadly similar (Figure 4; Supplementary Table S7). Evaluation Design had the highest high-risk proportion (Rater 1: 58/83, 69.9%; Rater 2: 63/83, 75.9%), followed by External Validity (42/83, 50.6%; 45/83, 54.2%). Outcome Reporting was most frequently judged low-risk (69/83, 83.1%; 70/83, 84.3%), while Task Definition was judged low-risk in 54/83 (65.1%) and 50/83 (60.2%) studies, respectively. Across 581 paired domain-level judgements, the reviewers agreed on 525 (90.4%), yielding a pooled Cohen’s κ of 0.837 (95% CI 0.772–0.895). Domain-specific κ values ranged from 0.703 for External Validity to 0.908 for Model Transparency. At the signaling-question level, agreement was 93.8% (1168/1245), with a pooled κ of 0.863 (95% CI 0.809–0.911; Supplementary Tables S8 and S10). 

### 3.8. Summary of Evidence Maturity

Collectively, the subtype taxonomy and cross-cutting analysis indicate that the strongest current empirical evidence lies in direct signal detection and information extraction, particularly DDI-related evaluation and EHR-based ADE/ADR identification, where representative quantitative results, such as F1-scores exceeding 0.90 [31] and AUROC values above 0.90 [75], have been reported under favorable experimental conditions. Operational pharmacovigilance workflow studies indicate that LLMs may also contribute substantially to literature screening, retrieval support, and medication-safety operations, with the RAG accuracy improving from 8.3% to 78.3% [45], representing the clearest single demonstration of what a structured system design can achieve within this domain. Document- and user-facing applications demonstrated practical value but raise significant concerns about reliability, completeness, and clinical appropriateness, which have not been resolved.

Overall, the current evidence supports a cautious and calibrated interpretation: LLMs are highly relevant as extraction, triage, retrieval, and documentation-support tools, although they are not yet reliable substitutes for expert pharmacovigilance judgment. The field is advancing rapidly, although the gap between benchmark performance and deployment readiness remains substantial. Bridging it will require prospective evaluation, external validation, and systematic attention to the specificity, hallucination, and reproducibility limitations currently inherent in the evidence base.

## 4. Discussion

### 4.1. Principal Findings

This review indicates that the literature on the applications of LLMs in ADRs, ADEs, and broader pharmacovigilance workflows has transcended the speculative phase, although its developmental maturity remains highly uneven across subdomains. The strongest empirical concentration is evident in direct signal-detection and information extraction tasks, specifically DDI-related evaluations and EHR-based ADE or ADR extractions [9,22,28,29]. Additional work is emerging in social media surveillance, literature screening, safety-document retrieval, ICSR-related processing, and expert-support workflows. Although the literature is expanding rapidly, much of this work remains proof-of-concept-oriented, benchmark-dependent, and only partially connected to real-world clinical or regulatory deployment.

A consistent technical reliability gradient was observed. Constrained information-extraction and classification tasks—such as NER, relation extraction, and structured screening—were more reproducible than open-ended tasks requiring pharmacological interpretation, causality assessment, or clinically accountable reasoning.

A third major finding is that current evidence supports the view that LLMs are better positioned as supervised support systems than autonomous pharmacovigilance agents. Across multiple subtypes, the literature repeatedly demonstrates that LLMs can help surface, structure, summarize, or pre-classify relevant information. However, the same literature shows that current models remain vulnerable to hallucination, false-positive overcalling, prompt sensitivity, and weak reproducibility [61,73].

### 4.2. Performance Disparities Between Extraction and Open-Ended Reasoning Tasks

One of the clearest patterns in the reviewed literature is that LLMs perform more convincingly when the task is narrow, bounded, and operationally constrained than when it requires open-ended clinical or pharmacological reasoning. This performance gradient can be understood through a three-tier task taxonomy that emerges from the current evidence. At the first tier sit well-structured extraction tasks, i.e., NER, relation extraction, and constrained classification, where the output space is defined, and reference standards exist. At the second tier sit retrieval and triage tasks, such as literature screening, signal pre-classification, and structured documentation support, where LLM outputs serve as upstream filters for human review. At the third tier sit interpretive judgment tasks, namely causality assessment, pharmacovigilance case adjudication, and regulatory reasoning, where outputs must be clinically accountable and legally defensible. The current empirical evidence is concentrated almost entirely within the first two tiers of this gradient, with the third tier remaining largely unsupported by robust, real-world evaluation. Representative first-tier performance was illustrated by Zhang et al. [31], who achieved an F1-score of 0.921 on ADR NER using error-correction prompting, and by Prioleau et al. (two independent studies from the same group) [39,40], who systematically compared prompting approaches for ADE detection in clinical text, demonstrating that structured inline annotation consistently outperformed entity-only extraction.

Conversely, third-tier tasks remain substantially beyond current LLM capability. For instance, Pandya et al. [55] found that ChatGPT produced ambiguous and inconsistent causality assessments compared with WHO–UMC and Naranjo-scale outputs. Montastruc et al. [11] concluded that LLMs lack the domain-specific reasoning reliability required for autonomous pharmacovigilance tasks. The underlying mechanistic explanation is fundamentally architectural: LLMs function as pattern-matching and text-generation engines that perform best when contextual constraints are rigid, and failure modes are easily verifiable. Conversely, causality assessment requires the integration of pharmacological plausibility, temporal relationships, counterfactual reasoning, and clinical context in manners that cannot be reduced to simple retrieval or classification tasks. This critical distinction (between tasks where LLMs extend human capacity and those where they merely simulate rather than replicate expert judgment) must serve as the primary lens through which operational deployment decisions are evaluated in pharmacovigilance.

### 4.3. Operational Importance of Specificity over Raw Sensitivity

A recurring operational problem was the combination of high sensitivity and low specificity in DDI and signal-evaluation studies [9,22,23]. The associated quantities must be distinguished precisely. The false-positive rate is FP/(FP + TN) and equals 1—specificity; the false discovery rate is FP/(TP + FP) and equals 1—positive predictive value (PPV). Thus, low specificity implies a high false-positive rate, while a low PPV implies a high false discovery rate; the terms are not interchangeable.

For operational deployment, sensitivity should therefore be reported alongside specificity, PPV, prevalence, and the projected number of false alerts. Even a modest false-positive rate can generate substantial adjudication work when applied to high-volume spontaneous-report, EHR, or social media streams. Systems should not be characterized as deployment-ready on sensitivity alone, and evaluation should quantify the downstream workload and clinical consequences of false alerts.

### 4.4. The Role and Structured Definition of Human Oversight

The three governance stages described here were derived inductively from recurring failures in the included studies rather than from a formal consensus process. Prompt sensitivity and incomplete configuration reporting motivated a design-and-validation stage [4,48,64]; hallucination, false-positive overcalling, and workflow confounding motivated structured human adjudication [2,9,61]; and version instability, weak external validation, and limited reproducibility motivated lifecycle monitoring [4,73]. Table 4 maps these evidence patterns to practical controls.

At deployment, outputs should remain auditable recommendations rather than final decisions. Controls should address automation bias and human error through independent verification, escalation of ambiguous outputs, provenance logging, and clearly as-signed clinical accountability. Lifecycle governance should document model and prompt updates, monitor data and performance drift, audit privacy and cybersecurity events, and define pre-specified suspension or rollback criteria when safety, specificity, or calibration thresholds are breached. These controls are evidence-informed proposals for future testing, not a validated governance standard.

### 4.5. Heterogeneity of Data Sources and Tasks in Pharmacovigilance

One of the strengths of the current literature is the ability to reflect the true heterogeneity of pharmacovigilance data. The reviewed studies span spontaneous reports, EHR notes, discharge summaries, social media text, patient narratives, biomedical literature, regulatory documents, and workflow documentation. A model that performs well on AE extracted from structured clinical text may not perform similarly on patient-generated narratives or social media content. Therefore, the literature argues against overgeneralized claims, such as “LLMs perform well in pharmacovigilance.” A more defensible claim is that LLMs demonstrate uneven but increasingly meaningful utility across specific pharmacovigilance subdomains.

Sensitive EHR and spontaneous-report narratives create substantial privacy and security constraints. External commercial APIs may be unsuitable for unredacted patient-safety data, whereas locally hosted open-weight models can reduce data egress but are not a cost-free solution. On-premise deployment requires accelerator hardware, secure storage, energy and maintenance budgets, model-operations infrastructure, cybersecurity monitoring, version control, and specialized engineering and pharmacovigilance expertise. These requirements may be prohibitive for smaller hospitals or resource-constrained pharmacovigilance centers. Deployment decisions must therefore balance privacy, performance, total cost of ownership, local technical capacity, and the availability of safe fallback workflows.

### 4.6. Current Trends and the Need for Methodological Standardization

Reporting was inconsistent for prompts, model/version identity, generation settings, rerun variability, failure analysis, data provenance, and external validation. Existing guidance provides an important foundation: TRIPOD+AI and PROBAST+AI address prediction-model reporting and appraisal [16,17]; TRIPOD-LLM addresses LLM-specific reporting, including model identity, prompting, evaluation, and human oversight [18]; CLAIM and CONSORT-AI address imaging and randomized AI studies [19,21]; and DECIDE-AI addresses early-stage clinical evaluation of AI decision-support systems [20].

Because these frameworks are broad or design-specific, they do not fully operationalize pharmacovigilance requirements such as adverse-event terminology, signal provenance, case-level audit trails, regulatory escalation, and workload consequences of false alerts. Table 5 therefore summarizes observed reporting deficits and candidate pharmacovigilance-specific items for future consensus development. It is not presented as a new reporting guideline. Formal prioritization, stakeholder consultation, and Delphi validation are required before any field-specific checklist can be recommended.

Future consensus work should explicitly map any pharmacovigilance-specific extension to TRIPOD-LLM and related guidance, retain only items that add domain-specific value, and test whether the proposed items improve reproducibility, auditability, and safety reporting in prospective studies.

### 4.7. Workflow Integration as a Near-Term Implementation Strategy

A particularly relevant implication of the reviewed literature is that near-term values will likely emerge less from autonomous clinical reasoning and more from workflow integration [2,3,45,47]. LLMs demonstrate maximum utility when embedded within structured human-in-the-loop review processes, where they function to minimize friction in information handling, retrieval, and preprocessing. A high sensitivity, moderate-specificity literature-screening tool may still save substantial effort if deployed as a first-pass triage assistant. This perspective aligns with broader evidence from health AI adoption: systems are often adopted more successfully when they augment existing workflows rather than attempt to outright replace expert judgment.

### 4.8. The Imperative for System-Level Design: RAG and Structured Workflows

The structural limitations identified across the reviewed literature, particularly the persistent low specificity in DDI evaluations [9,23] and high hallucination rates in clinical-note extraction [61,73], indicate that future advances in precision pharmacovigilance will depend less on scaling general base models and more on sophisticated system-level designs. The empirical evidence demonstrates that relying on standalone, ad hoc prompting is insufficient for safety-critical clinical environments.

To address the false-positive and hallucination issues observed in the main studies, future efforts must shift toward robust framework architectures, such as advanced RAG [61,72,76]. While several reviewed studies note that basic RAG improves factual grounding, the performance gaps in our synthesis highlight that drug-safety implementations must rigorously decouple retrieval failures from generation failures. In ADR detection, a retrieval failure occurs when the system cannot fetch the relevant patient history, directly capping sensitivity. Conversely, a generation failure occurs when the LLM hallucinates a causal link, thereby degrading specificity. Overcoming the limitations documented in this review requires dynamic knowledge-aware components, such as chunking strategies optimized for clinical narratives and semantic search over continuously updated medical ontologies.

Furthermore, mitigating the high false-positive rates observed in single-pass LLM evaluations [9] requires researchers to further explore agentic or multistep methodologies. Multiagent workflows, where distinct AI agents are assigned specific, bounded roles (e.g., extraction, cross-referencing, and critical-appraisal agents), can effectively simulate the internal peer-review processes of human pharmacovigilance teams. Grounding these technical architectures in the specific failure modes identified in current empirical studies can transition the field from experimental benchmarks to highly specific, clinically defensible tools necessary for precision medication safety.

### 4.9. Implications for Future Research

Four priorities follow from the evidence: prospective evaluation in realistic pharmacovigilance settings; reporting aligned with TRIPOD-LLM and future consensus-based pharmacovigilance extensions; system designs that separate retrieval from generation failures; and explicit distinction between constrained extraction performance and high-level clinical reasoning. Evaluations should also pre-specify human oversight, incident handling, and system-suspension criteria.

### 4.10. Limitations of This Review

This review has several limitations. The field is evolving rapidly, and studies published after the March 2026 search may alter the evidence landscape. Heterogeneity in study design, data sources, tasks, and outcomes precluded meta-analysis. Restriction to English-language reports may introduce language bias and limits conclusions about multilingual and global pharmacovigilance deployment. Several reports were abbreviated, early-stage, or conference-style publications, and uncertainty measures were frequently absent. The selection process used structured reviewer consensus, but independent pre-consensus eligibility decisions were not retained, preventing calculation of a valid screening-stage κ. Finally, the seven-domain evidence-limitation framework was adapted from established AI appraisal and reporting guidance but has not undergone independent prospective validation. Its results should be interpreted as a structured appraisal of recurring limitations rather than as a definitive validated RoB classification.

## 5. Conclusions

The current evidence does not establish LLMs as foundational or autonomous clinical systems for pharmacovigilance. Most included studies were retrospective, benchmark-based, or proof-of-concept evaluations. The clearest support is for constrained information extraction, classification, retrieval, and documentation tasks in which outputs remain readily verifiable by specialists.

The most plausible near-term role is therefore supervised, task-specific assistance embedded within human-in-the-loop workflows. LLMs may help surface and structure safety-relevant information, but they should not replace expert causality assessment, regulatory judgment, or accountability.

Concurrently, the literature consistently highlights several critical barriers to clinical adoption, including low specificity, hallucinated safety signals, prompt sensitivity, weak reproducibility, and a profound lack of prospective validation in real-world clinical settings. These constraints are fundamentally incompatible with the strict safety, traceability, and clinical accountability standards required in precision pharmacovigilance.

Progress will depend on prospective multi-site evaluation, external and multilingual validation, transparent reporting, reliable uncertainty and failure analysis, and governance that supports auditability, incident response, and system suspension. Until this evidence matures, LLMs should be deployed only in bounded roles with rigorous expert oversight.

## Figures and Tables

**Table 1:** Inclusion and exclusion criteria applied in the study-selection process.

Category	Criteria
Inclusion criteria	Published in English Addresses ADRs, ADEs, pharmacovigilance, medication safety, DDI-related safety analysis, or closely related drug-safety workflows Comprises an LLM, GAI model, foundation language model, or closely related generative language approach as a central methodological component Provides original empirical results, workflow-oriented evaluations, comparative benchmarks, directly relevant to ADR, ADE, or pharmacovigilance Published from January 2022 onward (core evidence base)
Exclusion criteria	Focuses on general medical chatbot use without clear relevance to ADR, ADE, drug safety, or pharmacovigilance workflows Centers on traditional ML or nongenerative NLP methods without an LLM component Too clinically peripheral, too broad, or too weakly aligned with the review scope after full-text assessment Lacks sufficient methodological detail for meaningful assessment after retrieval

**Table 2:** Representative quantitative results from included LLM studies. Sample or corpus size and measures of uncertainty are reported when available; NR indicates that the item was not reported or was not extractable from the available primary report. Numerical ranges reflect evaluation conditions and should not be interpreted as confidence intervals unless explicitly identified as such.

Ref.	Task/Data	Model/Design	Sample	Metric and Result	Uncertainty	Interpretation/Limitation
[14]	ADR NER; benchmark corpora	GPT-4 with error-correction prompting	NR	F1 = 0.921	NR	Benchmark-specific; external validation not reported
[15]	ADR knowledge probing	Frozen LLM hidden-state embeddings	NR	AUROC > 0.90	NR	Clinical utility not established
[16]	PV data retrieval; safety database	Context-augmented NLQ-to-SQL	NR	Accuracy 8.3% → 78.3%	NR	Context- and prompt-dependent
[17]	ADE NER; VAERS and social media	Deep learning and fine-tuned LLM ensemble	NR	Improved vs. single-model baselines	NR	Exact pooled estimate not reported in extracted record
[8]	DDI detection; AE reports	ChatGPT, Gemini, Claude	NR	High sensitivity; very low specificity	NR	Frequent false-positive interactions
[18]	Hospital-acquired PE detection; EMR	Three-module open-model framework	NR	Sensitivity 87.5–100%; specificity 94.9–98.9%	Ranges, not CIs	Retrospective evaluation; transportability uncertain

**Table 3:** Cross-cutting limitations identified across the included literature, summarizing the structural evidence gaps discussed in this section.

Limitation Category	Description	Affected Evidence	Representative Refs
Low specificity/false alerts	High sensitivity frequently co-occurred with low specificity, increasing review burden.	DDI and signal detection	[9,22,23]
Hallucinated outputs	Plausible but unsupported drugs, interactions, or causal claims.	Multiple task types	[61,73]
Prompt and version sensitivity	Performance changed with prompt wording, context, and model release.	NER, DDI, screening, QA	[4,48,64]
Narrow datasets	Small, retrospective, or task-specific corpora limited transportability.	Across task types	[73]
Weak external validation	Few multi-institutional, multilingual, or prospective evaluations.	Across task types	[8,73]
Missing uncertainty	Point estimates often lacked CIs, dispersion, or significance tests.	Quantitative evaluations	Supplementary Table S1
Workflow confounding	System-level interventions did not isolate the LLM contribution.	Human-in-the-loop workflows	[2]
Operational barriers	Compute, cybersecurity, model operations, and specialist staffing were underreported.	Local/open-weight deployment	Section 4.5
Limited reproducibility	Prompts, settings, reruns, and model versions were incompletely documented.	Across task types	[4,73,74]

**Table 4:** Evidence-linked governance controls for LLM-assisted pharmacovigilance. These controls are proposed for prospective evaluation and are not a validated standard.

Stage	Evidence Pattern	Required Controls	Escalation/Suspension Trigger
1. Design and validation	Prompt sensitivity; incomplete model and data reporting [4,48,64]	Versioned prompts/model; representative test sets; privacy and threat assessment; named owner	Validation below pre-specified sensitivity, specificity, PPV, fairness, or security thresholds
2. Human adjudication	Hallucination, false alerts, automation bias, workflow confounding [2,9,61]	Treat outputs as candidates; independent verification; provenance logging; override and escalation rules	Unverifiable source, ambiguous causality, repeated critical error, or reviewer disagreement
3. Lifecycle monitoring	Version instability, weak external validation, drift, and poor reproducibility [4,73]	Periodic audit; drift monitoring; change control; incident review; rollback capability	Threshold breach, material drift, cybersecurity incident, unannounced model change, or loss of auditability

**Table 5:** Observed reporting deficits in LLM-based pharmacovigilance studies and candidate items for future consensus development. The table is a synthesis of evidence gaps, not a validated reporting guideline.

Observed Deficit	Why It Matters in Pharmacovigilance	Related Guidance	Candidate Future-Consensus Item
Prompt and context not archived	Signal classification must be reproducible and auditable.	TRIPOD-LLM	Version-controlled system/user prompts, examples, and retrieval context
Model identity and updates unclear	Silent model changes can alter safety outputs.	TRIPOD-LLM; PROBAST+AI	Exact model/version, access date, update history, and rollback plan
Generation settings and rerun variance absent	Single-run estimates can conceal instability.	TRIPOD-LLM	All settings, repeated runs, variance, and sensitivity analyses
Failure cases incompletely reported	False alerts and missed signals have different clinical consequences.	DECIDE-AI; CONSORT-AI	Error taxonomy with FPR, FDR, PPV, missed-signal analysis, and workload impact
Case provenance and audit trail incomplete	Regulated PV decisions require traceable source evidence.	TRIPOD-LLM	Source-to-output provenance, reviewer actions, overrides, and escalation logs
Lifecycle governance absent	Data drift, cybersecurity events, and model updates can change risk.	PROBAST+AI; DECIDE-AI	Named accountability, monitoring thresholds, incident response, and suspension criteria

Note: Candidate items should be evaluated and refined by pharmacovigilance professionals, patients, regulators, methodologists, informaticians, and model developers through a formal consensus process.

## Data Availability

The original contributions presented in this study are included in the article and Supplementary Materials. Further inquiries can be directed to the corresponding author.

## Supplementary Materials

The following supporting information can be downloaded at: https://www.mdpi.com/article/10.3390/diagnostics16152435/s1; Table S1, Comprehensive characteristics and evaluation matrix for the 83 included empirical studies; Table S2, PRISMA 2020 screening summary distinguishing records, reports, and studies; Table S3, Itemized full-text exclusion reasons; Table S4, PRISMA 2020 checklist; Table S5, PICO framework; Table S6, Adapted seven-domain evidence-limitation framework with source mapping; Table S7, Paired pre-consensus domain judgements for both reviewers; Table S8, Inter-rater agreement results; Table S9, Full database search strategies and dates; and Table S10, Paired pre-consensus signaling-question-level judgements for both reviewers.

## Abbreviations

The following abbreviations are used in this manuscript:

AE	Adverse event
ADE	Adverse drug event
ADR	Adverse drug reaction
AI	Artificial intelligence
API	Application programming interface
AUROC	Area under the receiver operating characteristic curve
BERT	Bidirectional encoder representations from transformers
CI	Confidence interval
CLAIM	Checklist for Artificial Intelligence in Medical Imaging
CONSORT-AI	Consolidated Standards of Reporting Trials–Artificial Intelligence
DDI	Drug–drug interaction
DECIDE-AI	Developmental and Exploratory Clinical Investigations of Decision support systems driven by Artificial Intelligence
EHR	Electronic health record
EMR	Electronic medical record
FDR	False discovery rate
FDA	US Food and Drug Administration
FPR	False-positive rate
GAI	Generative artificial intelligence
LLM	Large language model
ML	Machine learning
NER	Named entity recognition
NLQ	Natural-language query
NLP	Natural-language processing
NR	Not reported
OSF	Open Science Framework
PPV	Positive predictive value
PRISMA	Preferred Reporting Items for Systematic Reviews and Meta-Analyses
PROBAST+AI	Prediction model Risk Of Bias Assessment Tool plus Artificial Intelligence
PV	Pharmacovigilance
RAG	Retrieval-augmented generation
RoB	Risk of bias
SQL	Structured query language
TRIPOD+AI	Transparent Reporting of a multivariable prediction model for Individual Prognosis Or Diagnosis plus Artificial Intelligence
TRIPOD-LLM	TRIPOD reporting guideline for studies using large language models
VAERS	Vaccine Adverse Event Reporting System
WHO–UMC	World Health Organization–Uppsala Monitoring Center
WoS	Web of Science
