# Supplementary material for: Large Language Models in Adverse Drug Reaction Detection and Pharmacovigilance: A Systematic Review of Current Applications, Challenges, and Future Directions
Source: Diagnostics (Basel). 2026 Aug 1;16(15):2435. doi: 10.3390/diagnostics16152435 (PMC13465311; doi:10.3390/diagnostics16152435)
Supplement: Supplementary file 1 [file diagnostics-16-02435-s001.zip › diagnostics-4430307-supplementary.pdf]

Supplementary Materials

Large Language Models in Adverse Drug Reaction Detection and Pharmacovigilance: A Systematic Review of Current Applications, Challenges, and Future Directions

Table S1. Comprehensive characteristics and evaluation matrix for the 83 included empirical studies. NR indicates information not reported or not extractable from the available primary report.

Abbreviations: AE, adverse event; ADE, adverse drug event; ADR, adverse drug reaction; CI, confidence interval; LLM, large language model; NR, not reported; PV, pharmacovigilance; RoB, risk of bias.

No.	Study (author, year)	Data source / dataset	LLM / model	PV task	Evaluation metric(s)	Sample / corpus size	Key finding / performance	Uncertainty	Key limitation(s)
1	Huang (2024.0)	mixed / multiple sources; custom curated corpus	Large LM(ChatGPT)	ADR recognition	Human rating	NR	5.07(4.26)	NR	High concern: Evaluation Design, External Validity; uncertainty NR
2	Kopacheva (2025.0)	clinical notes; internal hospital notes	Small LM(Bert)	ADE relation extraction	F1	NR	0.7	NR	High concern: Evaluation Design, External Validity, Comparator Strength; uncertainty NR
3	Sicard (2025.0)	adverse event reports; French pharmacovigilance database	Large LM(ChatGPT,Gemini,Claude)	DDI recognition	F1, Acc	NR	0.85, 0.66	NR	High concern: Evaluation Design, External Validity, Comparator Strength; uncertainty NR
4	Ong (2025.0)	EHR; multi-specialty clinical data	Small LM(RAG LM)	ADR, DDI recognition	F1, Acc	NR	0.59, 0.61	NR	High concern: Evaluation Design; uncertainty NR
5	Pais (2024.0)	clinical notes; custom curated corpus	Small LM(Flan-T5)	Prescription error recognition	Near-miss rate	NR	0.66 times lower	NR	uncertainty NR
6	Blotske (2025.0)	mixed / multiple sources; clinical text / databases	Large LM(GPT4o Mini)	DDI recognition	F1, Acc	NR	0.54, 0.61	NR	High concern: Evaluation Design, External Validity; uncertainty NR
7	Deng (2024.0)	social media posts; X (Twitter) dataset	Small LM(Roberta-Large)	ADE detetction	F1, Acc	NR	0.80, 0.97	NR	High concern: Evaluation Design, External Validity; uncertainty NR
8	Li (2024.0)	abstracts / full texts; pharmacovigilance literature	Large LM(GPT4)	Literature screening	F1, Acc	NR	0.82, 0.80	NR	uncertainty NR
9	Ma (2024.0)	abstracts / full texts (DILI dataset)	Small LM(PmC-LLaMA-7b)	Literature screening	F1, Acc	NR	0.97, 0.97	NR	uncertainty NR
10	Kulenovic (2025.0)	clinical notes; internal hospital notes	Large LM(ChatGPT)	DDI recognition	NR	NR	ChatGPT successfully suggested alternative drug classes to minimize ADRs and align with guidelines, improving the polypharmacy regimen.	NR	High concern: Evaluation Design, External Validity, Comparator Strength; uncertainty NR
11	Li (2025.0)	mixed / multiple sources; VAERS, social media dataset	Large LM(Fine-tuned GPT-3.5 with Rule 1)	relation extraction	F1, Precision, Recall	NR	0.97, 0.94, 1.0	NR	Unclear: Evaluation Design; uncertainty NR
12	Berkowitz (2025.0)	knowledge bases; ADR benchmark dataset	Large LM(Gemma2-9b-it)	classification	AUC-ROC	NR	0.957 (acute kidney injury), 0.902 (acute liver injury), 0.954 (acute myocardial infarction), 0.963 (gastrointestinal bleed)	NR	High concern: Evaluation Design, External Validity; uncertainty NR
13	Painter (2025.0)	regulatory documents; custom curated corpus (NLQ to SQL)	Large LM(GPT-4)	retrieval	Pass rate (Accuracy)	NR	0.783	NR	Rater concern: Evaluation Design, External Validity, Data Quality; uncertainty NR
14	Carpenter (2023.0)	mixed / multiple sources; custom curated corpus	Large LM(GPT-3)	NER + retrieval	F1, F2, Precision, Recall	NR	F1=0.770, F2=0.821 (alprazolam); F1=0.662, F2=0.735 (fentanyl) with drug name + Google filters	NR	High concern: Evaluation Design; uncertainty NR
15	Pandya (2024.0)	case reports; single clinical case	Large LM(ChatGPT)	decision support	NR	NR	ChatGPT provided ambiguous causality results compared to WHO-UMC and Naranjo scales, emphasizing the need for caution.	NR	High concern: Evaluation Design, External Validity, Data Quality; uncertainty NR
16	Xu (2024.0)	EHR; UK CPRD (6.6 million patients)	Large LM(ChatGPT)	decision support	Incidence Rate Ratio (IRR) with 95% CI	6.6 million patients; 16,901 pairs; 11,089 pairs	16,901 pairs with significant risk reduction; 11,089 pairs with significant risk increase	Reported (95% CI)	High concern: Evaluation Design, Comparator Strength
17	Benaïche (2025.0)	adverse event reports; Bayer internal pharmacovigilance cases	Large LM(MyGenAssist/ChatGPT-4 Turbo)	generation	time saved (%), response rate	NR	23.3% time saving (P<.001); response rate unchanged (42% vs 36%, P=.57)	Reported (p value)	High concern: Evaluation Design, External Validity
18	Zhang (2025.0)	mixed / multiple sources; custom curated corpus	Large LM(GPT-4-turbo)	NER	F1	NR	0.921 (partial match), 0.848 (exact match)	NR	Unclear: Task Definition; uncertainty NR
19	Gisladottir (2026.0)	drug labels; FDA labels	Large LM(GPT-4)	NER	F1, Precision, Recall	NR	macro-F1 semantic=0.9641, lenient=0.9095, strict=0.8311	NR	High concern: Evaluation Design; uncertainty NR

No.	Study (author, year)	Data source / dataset	LLM / model	PV task	Evaluation metric(s)	Sample / corpus size	Key finding / performance	Uncertainty	Key limitation(s)
20	Yazdani (2025.0)	social media posts; custom curated corpus (17,000 synthetic tweets)	Small LM(SyNORM-smm-L, CONORM with BERTweet-large)	NER	F1, Precision, Recall	NR	0.6339	NR	High concern: Evaluation Design, External Validity, Task Definition; uncertainty NR
21	Hosseini (2025.0)	clinical notes; psychiatric treatment data	Small LM(Fine-tuned Mistral-7B)	NER	F1, Precision, Recall	NR	0.8643, 0.896, 0.8643	NR	High concern: Evaluation Design, Task Definition; uncertainty NR
22	Jamil (2026.0)	mixed / multiple sources; cancer drug dataset	Large LM(LLaMA 3.1 8B with MMPSF)	NER + summarization	ROUGE-L, BLEU-4, METEOR, BERTScore F1, Clinical Evaluation Score, Factual Recall, Omission Rate	NR	ROUGE-L: 0.7078, BLEU-4: 0.4032, METEOR: 0.6401, BERT F1: 0.9672, CES: 4.18, FR: 0.92, OR: 0.19	NR	High concern: Evaluation Design; uncertainty NR
23	de Jesus (2025.0)	drug labels; custom curated corpus	Large LM(LLaMA 3.1 8b RAG Model)	generation	Adequacy (median), Clarity (median), Cosine Similarity	NR	Adequacy: 100 (IQR 15.0), Clarity: 95.0 (IQR 24.0), Similarity: 94.3 (IQR 5.3)	Reported (dispersion)	High concern: Evaluation Design, External Validity
24	Daluwatte (2024.0)	social media posts; global COVID-19 social media & web dataset	Small LM(PubmedBERT)	NER	Precision, Recall, F1	NR	0.76, 0.82, 0.79	NR	High concern: Evaluation Design, Comparator Strength; uncertainty NR
25	Montastruc (2023.0)	case reports; custom clinical cases	ChatGPT	QA / prompting	NR	NR	AI chatbots demonstrate potential in clinical pharmacology queries but currently cannot replace human experts due to accuracy and safety limitations.	NR	High concern: External Validity, Model Transparency; uncertainty NR
26	Li (2025.0)	mixed / multiple sources; VAERS, social media	Large LM(Fine-tuned GPT-3.5 with Rule 1)	relation extraction	F1, Precision, Recall	NR	0.97, 0.94, 1.0	NR	High concern: Evaluation Design; uncertainty NR
27	Hakim (2025.0)	NR	Small LM(mt5-xl)	generation	BLEU, AUROC, error rate	NR	BLEU: 0.39, AUROC: 0.80, clinical accuracy: 35%	NR	High concern: Evaluation Design; uncertainty NR
28	Koon (2025.0)	clinical notes; internal hospital notes (Discharge Summaries)	Large LM(Gemini 1.5 Pro)	relation extraction + classification	F1, Precision, Recall	NR	0.863, 0.943 (MIMIC-Unrestricted); hybrid model: 0.853 F1 on MIMIC-Unrestricted	NR	Unclear: Data Quality; uncertainty NR
29	Prioleau (2026.0)	clinical notes; ADE benchmark dataset	Small LM(RoBERTa-large)	NER + relation extraction	F1 (macro-F1)	NR	0.83043 (macro-F1, token classification)	NR	High concern: Evaluation Design, External Validity; uncertainty NR
30	Duan (2024.0)	social media posts; custom curated corpus (Semaglutide posts)	Large LM(GPT-4o Mini)	relation extraction	Accuracy	NR	0.7934 (side effects), 0.8685 (severity)	NR	High concern: Comparator Strength; uncertainty NR
31	Wu (2025.0)	drug labels; FDA Labeling Documents	Large LM(LLaMA 3.1-70B)	NER + classification	F1, Accuracy, Recall, Precision	NR	DILI F1=0.978, DICT F1=0.931, AE profiling F1=0.911	NR	High concern: Evaluation Design; uncertainty NR
32	Pariente (2024.0)	case reports; Custom pharmacovigilance questions	ChatGPT	QA / prompting	NR	NR	ChatGPT generally provided appropriate answers but lacked nuance and specific localized clinical context compared to PV center experts.	NR	High concern: External Validity; uncertainty NR
33	Wu (2026.0)	clinical notes; internal hospital notes	Small LM(LLaMA 3.1 8B - Error Analysis Prompting)	ADR extraction (classification of FP treatment and treatment-related toxicities)	F1	NR	1.000 (FP treatment), 0.965 (treatment-related toxicity)	NR	High concern: Evaluation Design, External Validity; uncertainty NR
34	Hu H. (2026.0)	knowledge bases; custom DDI benchmark	Large LM(Qwen2.5-Max-CoT)	DDI detection / drug combination risk classification	F1, AUC, AUPRC	NR	F1=0.971, AUC=0.982, AUPRC=0.974	NR	High concern: Evaluation Design; uncertainty NR
35	Bera A. (2023.0)	mixed / multiple sources; custom curated corpus	Small LM(DistilBERT-finetuned)	classification + NER + generation	F1, Acc, Precision, Recall	NR	F1=0.952, Acc=0.952, Precision=0.953, Recall=0.952	NR	High concern: Evaluation Design, External Validity, Comparator Strength; uncertainty NR
36	Jamil S. (2025.0)	mixed / multiple sources; cancer drug dataset	Small LM(T5 Large, GASCADE with DPO)	NER + summarization	ROUGE-1, ROUGE-2, ROUGE-L, BLEU, BERTScore, METEOR, Clinical Evaluation Score, Factual Recall, Omission Rate	NR	ROUGE-1: 0.3926, ROUGE-2: 0.1798, ROUGE-L: 0.3501, BERTScore: 0.4171, METEOR: 0.3678, Clinical Evaluation Score: 3.88, Factual Recall: 0.85, Omission Rate: 0.15	NR	High concern: Evaluation Design; uncertainty NR
37	Wang N. (2024.0)	EHR; patient portal messages	Large LM(GPT-4 Turbo)	generation	PPV (Positive Predictive Value)	NR	PPVraw: 0.45–0.47, PPVconsensus: 0.65–0.70, PPVconsensus+rare: 0.71–0.72	NR	High concern: Evaluation Design, External Validity, Comparator Strength; uncertainty NR
38	Liu J. (2025.0)	social media posts; X (Twitter) Japan	NLP	classification	NR	NR	NLP analysis of social media identified key themes and sentiments driving HPV vaccination hesitancy in Japan.	NR	High concern: Evaluation Design, External Validity; uncertainty NR
39	Ito T. (2025.0)	EHR; Japanese EHRs	Small LM(T5)	classification	Precision, Recall, F1	NR	0.83, 0.25, 0.38	NR	High concern: Evaluation Design, External Validity, Comparator Strength; uncertainty NR
40	Ren X. (2026.0)	mixed / multiple sources; custom curated corpus	Large LM(DeepSeek-V3)	NER + relation extraction	F1, Acc, AE	NR	Drug entity recognition/standardization F1=0.960/0.960, Symptom recognition/standardization F1=0.862/0.818, Causal relationship mining F1=0.825, Causality AE=1.047, Acc=0.541	NR	High concern: Evaluation Design, Model Transparency; uncertainty NR

No.	Study (author, year)	Data source / dataset	LLM / model	PV task	Evaluation metric(s)	Sample / corpus size	Key finding / performance	Uncertainty	Key limitation(s)
41	Abate A. (2025.0)	adverse event reports; COVID-19 ICSRs	Large LM(ChatGPT GPT-3.5-turbo-16k)	AEFI causality assessment classification + decision support	percent agreement, F1, accuracy, sensitivity, specificity	NR	agreement 71% (ChatGPT), 53% (Gemini); F1 0.82 (ChatGPT), 0.63 (Gemini); accuracy 0.91 (ChatGPT), 0.91 (Gemini)	NR	High concern: Evaluation Design, External Validity; uncertainty NR
42	Dou Y. (2025.0)	knowledge bases; Chinese medication knowledge base	Small LM(BioMed-DeBERTa with knowledge augmentation)	NER (ADR extraction + ADR normalization)	F1 (μF1), Precision (μP), Recall (μR)	NR	μF1: 0.855 (ADR extraction, Reported Adverse Effects), F1: 0.571 (ADR normalization), μF1: 0.879 (ADR extraction, Cadec)	NR	High concern: Evaluation Design, External Validity, Data Quality; uncertainty NR
43	Bertalan Á. (2025.0)	knowledge bases; custom clinical data	Large LM(GPT-4o)	decision support	Spearman correlation	NR	0.463 (Expert 1), 0.496 (Expert 2)	NR	Rater concern: Model Transparency, Data Quality; uncertainty NR
44	Sridharan K. (2024.0)	regulatory documents; USFDA patient communications	LLMs	generation	NR	NR	LLMs effectively rewrote and significantly improved the readability scores of complex FDA patient safety communications.	NR	High concern: Evaluation Design, External Validity; uncertainty NR
45	Osheba A. (2025.0)	mixed / multiple sources; custom pharmacy dataset	Large LM(LLaMA 3.1)	DDI recognition + decision support	Acc	NR	0.83 (overall), 0.88 (interacting), 0.79 (non-interacting)	NR	High concern: Evaluation Design, External Validity, Comparator Strength; uncertainty NR
46	Wang Z. (2025.0)	adverse event reports; Vaccine AE dataset	Large LM(GPT-4)	classification	F1, Accuracy, Precision, Recall	NR	Composite F1: higher than RxNorm; Ingredient accuracy: 0.99; Strength F1 (NA excluded): 0.87	NR	High concern: Evaluation Design, External Validity; uncertainty NR
47	De Vito G. (2025.0)	clinical notes; hospital clinical data	Large LM(GPT-4o, Gemma 3 12B, Claude Sonnet 4)	classification + decision support	Precision, Recall, F1, Accuracy	NR	GPT-4o: F1=0.9869 (synthetic), F1=0.9963 (real-world); Gemma 3: F1=0.9904 (synthetic), F1=0.9928 (real-world); Claude Sonnet: F1=0.9950 (synthetic), F1=0.9964 (real-world)	NR	Unclear: Comparator Strength; uncertainty NR
48	Li H. (2025.0)	mixed / multiple sources; Chinese ADR dataset	LLM	relation extraction	NR	NR	Enhanced ADR relation extraction using Chain of Thought (CoT) prompting across different data resource settings.	NR	High concern: Evaluation Design, Task Definition; uncertainty NR
49	Socrates V. (2025.0)	EHR; retrospective cohort EHR	Large LM(GPT-4o)	decision support + classification	F1, PPV, NPV	NR	Step1 F1=0.86 (PPV=0.83, NPV=0.93); Step2 F1=0.58 (PPV=0.47, NPV=0.93)	NR	High concern: Evaluation Design, External Validity; uncertainty NR
50	Bakouny Z. (2025.0)	clinical notes; oncology EHR / notes	Large LM(LLaMA3-8B fine-tuned via QLoRA)	ADR detection classification	F1, Precision, Recall	NR	F1: 87.4% (Twitter), 85.5% (TwiMed-Twitter), 92.4% (CADEC)	NR	High concern: Evaluation Design, External Validity, Model Transparency; uncertainty NR
51	Li Y. (2024.0)	adverse event reports; VAERS (influenza)	AE-GPT	NER	NR	NR	AE-GPT fine-tuned on surveillance reports showed superior performance in extracting influenza vaccine AEs.	NR	High concern: Evaluation Design, External Validity, Data Quality; uncertainty NR
52	Javed M. (2025.0)	social media posts; online public forums / social media	Small LM(CT-BERT V2), Large LM(GPT-4o)	classification + summarization	Accuracy	NR	0.93	NR	High concern: Evaluation Design, Comparator Strength; uncertainty NR
53	Sheikh M.S. (2024.0)	knowledge bases; custom clinical scenarios (CKD patients)	Large LM(ChatGPT-4)	classification	Concordance %, Cohen's Kappa	NR	81.4%, κ=0.83	NR	High concern: Evaluation Design, External Validity, Data Quality; uncertainty NR
54	Qiu Y. (2026.0)	mixed / multiple sources; ADR benchmark dataset	LLM	NER	NR	NR	Dynamic knowledge-aware integration significantly improved the accuracy of LLMs in recognizing ADR entities.	NR	Unclear: External Validity; uncertainty NR
55	Zou H. (2025.0)	mixed / multiple sources; custom ADR corpus	LLM	classification	NR	NR	Knowledge-augmented framework (RAG) enhanced the overall reliability and accuracy of ADR analysis tasks.	NR	Rater concern: Data Quality; uncertainty NR
56	He C. (2025.0)	clinical notes; medical symptom datasets	LLMs	classification	NR	NR	Task as Context' prompting strategy significantly improved the accuracy of medical symptom coding.	NR	Unclear: Evaluation Design; uncertainty NR
57	Guillot J. (2024.0)	clinical notes; oncology EHR (CAR-T patients)	Large LM(GPT-4)	NER + classification	Acc	NR	0.64	NR	High concern: Evaluation Design; uncertainty NR
58	Bužančić I. (2024.0)	case reports; clinical scenarios	Large LM(ChatGPT-4)	decision support	Agreement rate, Cohen's Kappa	NR	95% agreement rate, κ=0.200	NR	Unclear: Evaluation Design, Model Transparency; uncertainty NR
59	Yin H. (2025.0)	social media posts; social media ADR dataset	Small LM(ChatGLM3-6B with LoRA, LLMADR)	NER + relation extraction + classification	F1, Acc, Precision, Recall	NR	Classification internal F1: 96.03%, external F1: 77.70%; ADR Extraction internal F1: 58.35%, external F1: 49.02%	NR	Rater concern: Evaluation Design, External Validity, Task Definition; uncertainty NR
60	Dezoteux F. (2025.0)	EHR; internal hospital notes	LLM	classification	NR	NR	The privacy-preserving LLM successfully automated the detection of drug hypersensitivity reactions within a secure hospital environment.	NR	High concern: Evaluation Design, External Validity; uncertainty NR
61	Zheng Y. (2025.0)	EHR; real-world dispensing data	LLM	classification	NR	NR	The LLM showed comparable or superior performance to standard RxNorm mapping rules for detecting medication dispensing errors.	NR	Unclear: Evaluation Design; uncertainty NR
62	Gitman V. (2025.0)	abstracts / full texts; custom literature	Large LM(GPT-4)	retrieval	Jaccard similarity	NR	Jaccard similarity: 0.17 (exact), 0.23	NR	Unclear: Evaluation Design;

No.	Study (author, year)	Data source / dataset	LLM / model	PV task	Evaluation metric(s)	Sample / corpus size	Key finding / performance	Uncertainty	Key limitation(s)
		dataset			(exact/semantic), proportion of omitted keywords recreated		(semantic); proportion: 0.49 (exact), 0.71 (semantic)		uncertainty NR
63	Kim M. (2025.0)	mixed / multiple sources; ADR benchmark dataset	Transformer-based models, LLMs	classification	NR	NR	Fine-tuned smaller transformer models demonstrated competitive cross-drug ADR detection capabilities when benchmarked against zero-shot LLMs.	NR	uncertainty NR
64	Prioleau H. (2025.0)	clinical notes; ADE benchmark dataset	Small LM(LLaMA-3.1 8B)	NER	Lenient F1, Precision, Recall	NR	Drug F1: 0.525, ADE F1: 0.115 (entity-only, zero-shot)	NR	High concern: Evaluation Design; uncertainty NR
65	Prioleau H. (2025.0)	clinical notes; ADE benchmark dataset	LLMs	NER	NR	NR	Compares prompt strategies, showing how entity-only vs. inline approaches affect LLM performance in ADE detection.	NR	High concern: Evaluation Design; uncertainty NR
66	Elbiach O. (2025.0)	clinical notes; patient narratives corpus	Large LM(GPT-4o-mini)	NER	Relaxed F1, Strict F1	NR	Relaxed F1: 79.06% (CADEC), 66.80% (SMM4H); Strict F1: 71.69% (CADEC), 59.23% (SMM4H)	NR	uncertainty NR
67	Burnette H. (2024.0)	case reports; custom clinical scenarios (irAEs)	Large LM(ChatGPT GPT-4)	decision support	Likert scale mean score (accuracy, completeness)	NR	Accuracy mean 3.87/4, Completeness mean 3.83/4	NR	Unclear: Model Transparency; uncertainty NR
68	Painter J.L. (2023.0)	regulatory documents; drug safety documentation	Large LM(GPT-4)	retrieval	Likert accuracy scale (1-6), Likert completeness scale (1-3)	NR	Mean accuracy: 4.750, Median accuracy: 5.750; Mean completeness: 2.227, Median completeness: 2.500	NR	High concern: Evaluation Design; uncertainty NR
69	Sun V.H. (2024.0)	EHR; hospitalized patient EHR	Large LM(LLM with RAG)	classification	Sensitivity, Specificity	NR	Sensitivity: 94.2%, Specificity: 92.5%	NR	uncertainty NR
70	Wakabayashi S. (2025.0)	clinical notes; Nursing Records	Large LM(ChatGPT-4)	classification	NR	NR	ChatGPT successfully categorized and extracted pharmaceutical safety information directly from unstructured nursing records.	NR	High concern: Evaluation Design, External Validity, Comparator Strength; uncertainty NR
71	Ying L. (2024.0)	drug labels; FDA labels	Large LM(ChatGPT, gpt-3.5-turbo-16k)	summarization	Cosine similarity (BERT-based)	NR	87.99% of pairs with similarity >0.8; 59.59% with similarity >0.9	NR	High concern: Evaluation Design, External Validity; uncertainty NR
72	Khademi S. (2025.0)	clinical notes; ED triage notes	Fine-tuned LLMs	NER	NR	NR	Fine-tuned LLMs accurately extracted vaccine mentions from noisy emergency department triage notes, enhancing active surveillance.	NR	High concern: External Validity; uncertainty NR
73	Vismaya S. (2025.0)	clinical notes; psychiatric clinical text	LLM	classification	NR	NR	The In-Context Pruning framework efficiently isolated relevant psychiatric adverse effects from complex narratives.	NR	Unclear: Evaluation Design, External Validity, Data Quality; uncertainty NR
74	Hu H. (2025.0)	abstracts / full texts; DDI extraction dataset	CoTEL-D3X	relation extraction	NR	NR	CoTEL-D3X achieved state-of-the-art results in extracting complex DDI triplets using chain-of-thought fine-tuning.	NR	uncertainty NR
75	Alshehri B.M.J. (2024.0)	knowledge bases; DDI dataset	Small LM(DistilBERT)	DDI recognition	F1, Acc	NR	0.8899 (DDI), 0.9387 (PDI), Acc: 0.906 (DDI), 0.955 (PDI)	NR	High concern: External Validity, Data Quality, Task Definition; uncertainty NR
76	Chandra M. (2025.0)	social media posts; custom psychiatric queries	Large LM(Claude 3 Opus, OpenBioLLM-Llama3-70B)	ADR detection + classification + harm reduction response generation	F1, Acc, AlignScore, GPT-4o Score, Actionability Score	NR	F1: 76.44 (ADR detection, Claude 3 Opus zero-shot), F1: 76.69 (multiclass, Llama3.1-405B 5-shot), GPT-4o Score: 70.86 (OpenBioLLM), Actionability: 0.44 (OpenBioLLM)	NR	High concern: Evaluation Design, External Validity; uncertainty NR
77	Fu W. (2025.0)	mixed / multiple sources; custom ADR corpus	LLM	classification + NER	NR	NR	Combining data augmentation with instruction fine-tuning significantly boosted ADR detection capabilities in resource-limited settings.	NR	High concern: Evaluation Design, External Validity, Data Quality; uncertainty NR
78	Zhang R. (2025.0)	mixed / multiple sources; cross-domain DDI data (TCM + Western)	Hybrid LLM-BiNN	relation extraction	NR	NR	The hybrid model successfully bridged domain gaps, annotating DDIs between Traditional Chinese Medicine (East) and Western medicine.	NR	uncertainty NR
79	Elbiach O. (2025.0)	mixed / multiple sources; Twitter + clinical notes	LLMs	NER	NR	NR	Provided a comprehensive benchmark showing varying LLM efficacies across noisy social media vs. structured clinical texts.	NR	uncertainty NR
80	Liu Q. (2025.0)	abstracts / full texts; Chinese literature	Generative AI	generation	NR	NR	The platform automated the generation of structured ADR reports directly from unstructured literature, standardizing workflows.	NR	High concern: Evaluation Design, External Validity; uncertainty NR
81	Kim J. (2025.0)	knowledge bases; custom OTC/herbal pairs	LLM	classification	NR	NR	The LLM effectively stratified risk levels for DDIs involving OTCs and herbal supplements, aligning closely with expert consensus.	NR	High concern: Evaluation Design, Data Quality, Task Definition; uncertainty NR
82	Wei (2026.0)	EHR; multicenter IBD EHRs	Large LM(HYBRID + LR ensemble LLM pipeline)	NER + relation extraction + classification	AUC, F1, Recall, Precision	NR	AUC 0.809 (CD), 0.828 (UC); F1 0.577 (CD), 0.545 (UC)	NR	High concern: Evaluation Design, External Validity, Comparator

No.	Study (author, year)	Data source / dataset	LLM / model	PV task	Evaluation metric(s)	Sample / corpus size	Key finding / performance	Uncertainty	Key limitation(s)
									Strength; uncertainty NR
83	Dietrich (2025.0)	mixed / multiple sources; NER benchmark datasets	Small LM(T5-Base fine-tuned)	NER + relation extraction	partial F1, partial recall	NR	highest F1 for product combination entities compared to all other models	NR	High concern: Evaluation Design; uncertainty NR

Table S2. PRISMA 2020 screening summary distinguishing database records, full-text reports, and unique included studies.

PRISMA unit	Stage	PubMed	Scopus	Web of Science	Total
Records	Identified before deduplication	107	404	151	662
Records	Exact bibliographic duplicates removed	—	—	—	150
Records	Unique records screened	—	—	—	512
Records	Excluded after title/abstract screening	—	—	—	298
Reports	Sought for retrieval	—	—	—	214
Reports	Not retrieved	—	—	—	0
Reports	Assessed for eligibility	—	—	—	214
Reports	Excluded after full-text assessment	—	—	—	131
Studies	Unique empirical studies included	—	—	—	83

Table S3. Reasons for exclusion at full-text assessment (n = 131 reports).

Reason for exclusion	n	Operational definition
Contextual reviews, perspectives, or commentaries	48	Used only for narrative framing; no original eligible empirical LLM-PV evaluation
Background or comparator-only reports	34	No direct empirical contribution to an eligible LLM pharmacovigilance task
Methodological or benchmark reports without direct PV application	12	Method was not evaluated in an ADR/ADE/drug-safety context
LLM not the primary methodological component	5	LLM contribution was incidental or not separable from the primary method
Overlapping or derivative reports	32	Distinct publications based on the same underlying dataset/cohort or a derivative analysis already represented, without a separable eligible contribution
Total	131	All full-text exclusion categories were mutually exclusive

Table S4. PRISMA 2020 checklist, indicating the manuscript section in which each reporting item is addressed.

Section and Topic	Item #	Checklist Item	Location Where Item Is Reported
**TITLE**			Title page — manuscript title explicitly states 'A Systematic Review'
Title	1	Identify the report as a systematic review.	Title page — manuscript title explicitly states 'A Systematic Review'
**ABSTRACT**			Abstract (structured): Background/Objectives, Methods, Results, Conclusions
Abstract	2	See the PRISMA 2020 for Abstracts checklist.	Abstract (structured): Background/Objectives, Methods, Results, Conclusions — see PRISMA 2020 for Abstracts checklist
**INTRODUCTION**			Section 1 (Introduction), ¶1–5 — gap statement and rationale for LLM-focused PV review
Rationale	3	Describe the rationale for the review in the context of existing knowledge.	Section 1—background, evidence gap, and rationale for an LLM-focused pharmacovigilance review
Objectives	4	Provide an explicit statement of the objective(s) or question(s) the review addresses.	End of Section 1—explicit review objectives and research questions (RQ1–RQ5)
**METHODS**			Section 2.4 (Eligibility Criteria) — PICO-based inclusion/exclusion criteria; Table 1
Eligibility criteria	5	Specify the inclusion and exclusion criteria for the review and how studies were grouped for the syntheses.	Section 2.4; Table 1; Supplementary Table S5—eligibility criteria and PICO scope
Information sources	6	Specify all databases, registers, websites, organisations, reference lists and other sources searched or consulted to identify studies. Specify the date when each source was last searched or consulted.	Section 2.3; Supplementary Table S9—PubMed, Scopus, and Web of Science, with coverage and final search date
Search strategy	7	Present the full search strategies for all databases, registers and websites, including any filters and limits used.	Section 2.3; Supplementary Table S9—complete database-specific strings, coverage, and final search date
Selection process	8	Specify the methods used to decide whether a study met the inclusion criteria of the review, including how many reviewers screened each record and each report retrieved, whether they worked independently, and if applicable, details of automation tools used in the process.	Section 2.5—two reviewers (T.Y.K., W.-S.O.) used structured consensus; independent pre-consensus selection decisions were not retained
Data collection process	9	Specify the methods used to collect data from reports, including how many reviewers collected data from each report, whether they worked independently, any processes for obtaining or confirming data from study investigators, and if applicable, details of automation tools used in the process.	Section 2.6—structured extraction matrix, reviewer checking, and handling of missing information
Data items	10a	List and define all outcomes for which data were sought. Specify whether all results that were compatible with each outcome domain in each study were sought (e.g. for all measures, time points, analyses), and if not, the methods used to decide which results to collect.	Section 2.6; Supplementary Table S1—study, dataset, model, task, sample/corpus size, metrics, performance, uncertainty, findings, and limitations
	10b	List and define all other variables for which data were sought (e.g. participant and intervention characteristics, funding sources). Describe any assumptions made about any missing or unclear information.	Section 2.8; Supplementary Tables S6–S8 and S10—adapted appraisal domains, signalling questions, paired ratings, and reliability estimates
Study risk of bias assessment	11	Specify the methods used to assess risk of bias in the included studies, including details of the tool(s) used, how many reviewers assessed each study and whether	Section 2.8; Supplementary Tables S6–S8 and S10—adapted seven-domain framework, two independent reviewers, Cohen's κ, and paired ratings

		they worked independently, and if applicable, details of automation tools used in the process.	
Effect measures	12	Specify for each outcome the effect measure(s) (e.g. risk ratio, mean difference) used in the synthesis or presentation of results.	Section 2.6—study-level performance metrics were extracted as reported; no common effect measure was imposed
Synthesis methods	13a	Describe the processes used to decide which studies were eligible for each synthesis (e.g. tabulating the study intervention characteristics and comparing against the planned groups for each synthesis (item #5)).	Section 2.7—studies were grouped by task/application layer for narrative synthesis; no meta-analysis was undertaken
	13b	Describe any methods required to prepare the data for presentation or synthesis, such as handling of missing summary statistics, or data conversions.	Section 2.6—missing or unavailable values were recorded as not reported; no effect-size conversion or imputation was performed
	13c	Describe any methods used to tabulate or visually display results of individual studies and syntheses.	Sections 2.7 and 3; Table 2; Supplementary Table S1—tabular and narrative presentation by task and application layer
	13d	Describe any methods used to synthesize results and provide a rationale for the choice(s). If meta-analysis was performed, describe the model(s), method(s) to identify the presence and extent of statistical heterogeneity, and software package(s) used.	Section 2.7—narrative synthesis was selected because methodological and outcome heterogeneity precluded quantitative pooling
	13e	Describe any methods used to explore possible causes of heterogeneity among study results (e.g. subgroup analysis, meta-regression).	Sections 3.2–3.6 and 4.2–4.6—heterogeneity examined qualitatively by task, model/system design, data source, and evaluation setting
	13f	Describe any sensitivity analyses conducted to assess robustness of the synthesized results.	Not applicable—no statistical synthesis or sensitivity analysis was conducted
Reporting bias assessment	14	Describe any methods used to assess risk of bias due to missing results in a synthesis (arising from reporting biases).	Sections 2.7–2.8 and 4.10—formal publication-bias analysis was not appropriate for the narrative synthesis; reporting limitations were assessed descriptively
Certainty assessment	15	Describe any methods used to assess certainty (or confidence) in the body of evidence for an outcome.	Section 2.8; Supplementary Tables S6–S8 and S10—adapted evidence-limitation appraisal; no GRADE certainty rating was assigned
**RESULTS**			Section 3.1; Figure 1 (PRISMA Flow Diagram) — 662 identified → 83 included
Study selection	16a	Describe the results of the search and selection process, from the number of records identified in the search to the number of studies included in the review, ideally using a flow diagram.	Section 3.1; Figure 1; Supplementary Tables S2–S3—662 records, 214 reports assessed, and 83 unique studies included
	16b	Cite studies that might appear to meet the inclusion criteria, but which were excluded, and explain why they were excluded.	Sections 3.1–3.4; Table 2; Supplementary Table S1—citations and characteristics for all 83 included studies
Study characteristics	17	Cite each included study and present its characteristics.	Sections 3.1–3.7; Table 2; Supplementary Table S1—study-level characteristics, results, uncertainty when reported, and limitations
Risk of bias in studies	18	Present assessments of risk of bias for each included study.	Section 3.7; Figure 4; Supplementary Tables S7–S8 and S10—paired domain and signalling-question ratings and reliability results
Results of individual studies	19	For all outcomes, present, for each study: (a) summary statistics for each group (where appropriate) and (b) an effect estimate and its precision (e.g. confidence/credible interval), ideally using structured tables or plots.	Sections 3.2–3.4; Table 2; Supplementary Table S1—study-level results, sample/corpus size, and uncertainty where reported
Results of syntheses	20a	For each synthesis, briefly summarise the characteristics and risk of bias among contributing	Section 3—narrative synthesis by task and application layer; Table 2 and Supplementary Table S1

		studies.	
	20b	Present results of all statistical syntheses conducted. If meta-analysis was done, present for each the summary estimate and its precision (e.g. confidence/credible interval) and measures of statistical heterogeneity. If comparing groups, describe the direction of the effect.	Sections 3.5 and 4.2–4.6—heterogeneity interpreted by task type, model/system design, data source, and setting
	20c	Present results of all investigations of possible causes of heterogeneity among study results.	Not applicable—no sensitivity analyses were conducted
	20d	Present results of all sensitivity analyses conducted to assess the robustness of the synthesized results.	Sections 3.6 and 4.10—reporting limitations, absent uncertainty, and preprint/early-stage evidence discussed qualitatively
Reporting biases	21	Present assessments of risk of bias due to missing results (arising from reporting biases) for each synthesis assessed.	Sections 3.6 and 4.10; Table 3—risk of missing or selectively reported results discussed qualitatively
Certainty of evidence	22	Present assessments of certainty (or confidence) in the body of evidence for each outcome assessed.	Section 3.7; Figure 4; Supplementary Tables S7–S8 and S10—structured evidence-limitation appraisal results; no GRADE certainty rating
**DISCUSSION**			Section 4.1 (Principal Findings) — interpretation relative to prior NLP-PV reviews
Discussion	23a	Provide a general interpretation of the results in the context of other evidence.	Sections 4.1–4.9—interpretation in relation to the included evidence and relevant AI reporting/appraisal guidance
	23b	Discuss any limitations of the evidence included in the review.	Sections 3.6 and 4.10—limitations of the evidence base, including reporting, external validation, and workflow confounding
	23c	Discuss any limitations of the review processes used.	Section 4.10—English-language restriction, consensus screening without retained independent decisions, rapidly evolving evidence, and adapted appraisal framework
	23d	Discuss implications of the results for practice, policy, and future research.	Sections 4.7–4.9 and Section 5—implications for supervised deployment, governance, and future research
**OTHER INFORMATION**			Section 2.1 — OSF pre-registration: https://osf.io/B8u9e (embargoed until 30 September 2026)
Registration and protocol	24a	Provide registration information for the review, including register name and registration number, or state that the review was not registered.	Section 2.1—OSF protocol URL and embargo status
	24b	Indicate where the review protocol can be accessed, or state that a protocol was not prepared.	Section 2.1—protocol access information
	24c	Describe and explain any amendments to information provided at registration or in the protocol.	Section 2.1—no amendments to the registered protocol were reported
Support	25	Describe sources of financial or non-financial support for the review, and the role of the funders or sponsors in the review.	Funding section—financial support and funder information
Competing interests	26	Declare any competing interests of review authors.	Conflicts of Interest section—authors declare no competing interests
Availability of data, code and other materials	27	Report which of the following are publicly available and where they can be found: template data collection forms; data extracted from included studies; data used for all analyses; analytic code; any other materials used in the review.	Supplementary Tables S1–S10, including paired pre-consensus domain- and signalling-question-level ratings

Table S5. PICO (Population, Intervention, Comparator, Outcome) framework defining the eligibility scope of this review.

Component	Definition	Operationalization in This Review
**Population (P)**	Pharmacovigilance and medication-safety-related contexts	Studies involving patient-level or text-based safety data sources, including electronic health records (EHRs), clinical notes, spontaneous reports, and social media data
**Intervention (I)**	Application of large language models (LLMs) or generative AI systems	Use or evaluation of general-purpose foundation models (e.g., GPT-based systems) or domain-adapted/fine-tuned LLMs as a central methodological component in medication-safety-related tasks
**Comparator (C)**	Variable across studies	Included traditional NLP models, rule-based systems, or human expert evaluation; comparators were not consistently defined across all studies
**Outcome (O)**	Performance and practical utility measures	Quantitative metrics (e.g., accuracy, sensitivity, specificity, F1-score) and qualitative or workflow-related outcomes (e.g., usability, clinical relevance, efficiency)

Table S6. Adapted seven-domain evidence-limitation framework, signalling questions, source-framework mapping, and adaptation rationale.

Domain	Signalling questions	Source framework(s)	Adaptation rationale
Data Quality	1. Representative real-world PV data?<br>2. Reliable annotation described?<br>3. Preprocessing reported?	PROBAST+AI; TRIPOD+AI	Captures representativeness, annotation validity, and data-processing transparency
Task Definition	1. Task clearly defined and reproducible?<br>2. Case eligibility criteria specified?	TRIPOD-LLM; TRIPOD+AI	Separates reproducible extraction/classification tasks from underspecified prompts
Model Transparency	1. Model and prompting described?<br>2. Fine-tuning and generation settings reported?	TRIPOD-LLM; CLAIM	Addresses model/version, prompt, parameter, and update transparency
Evaluation Design	1. Appropriate evaluation data/reference standard?<br>2. External validation performed?	PROBAST+AI; DECIDE-AI	Assesses internal evaluation integrity and independent validation
Comparator Strength	1. Strong baseline or human comparator?<br>2. Comparator methods described?	CONSORT-AI; DECIDE-AI	Tests whether claimed gains are attributable to the LLM/system
Outcome Reporting	1. Metrics appropriate and defined?<br>2. Results complete, including uncertainty/failures?	TRIPOD-LLM; TRIPOD+AI	Captures selective point estimates, absent uncertainty, and incomplete failure analysis
External Validity	1. Multiple data sources/settings/languages evaluated?<br>2. Generalizable to real-world PV?	PROBAST+AI; TRIPOD-LLM	Assesses transportability across institutions, languages, populations, and workflows
Decision rule	Low: all Yes<br>High: any No<br>Unclear: otherwise	Prespecified adaptation	Applied consistently to both independent raters before reliability analysis

Table S7. Paired pre-consensus domain-level judgements for both independent reviewers across all 83 included studies. Each cell reports Rater 1 / Rater 2.

Low = all signalling questions answered Yes; High = at least one No; Unclear = otherwise. Green indicates agreement on Low, orange agreement on High, gray agreement on Unclear, and yellow a discordant domain judgement.

Study ID	Author	DQ	TD	MT	ED	CS	OR	EV
FINAL_0001	Huang	Low / Low	Unclear / Unclear	Unclear / Unclear	High / High	Low / Low	Low / Low	High / High
FINAL_0004	Kopacheva	High / High	High / High	High / High	High / High	High / High	Low / Low	High / High
FINAL_0007	Sicard	Low / Low	Low / Low	Low / Low	High / High	High / High	Low / Low	High / High
FINAL_0008	Ong	Low / Low	Low / Low	Low / Low	High / High	Low / Low	Low / Low	Unclear / Unclear
FINAL_0018	Pais	Low / Low	Low / Low	Low / Low	Low / Low	Low / Low	Low / Low	Low / Low
FINAL_0020	Blotske	Low / Low	Low / Low	Low / Low	High / High	Unclear / Unclear	Low / Low	High / High
FINAL_0023	Deng	Low / Low	Low / Low	Low / Low	High / High	Low / Low	Low / Low	High / High
FINAL_0029	Li	Low / Low	Low / Low	Low / Low	Low / Low	Low / Low	Low / Low	Low / Low
FINAL_0033	Ma	Low / Low	Low / Unclear	Low / Low	Low / High	Low / Low	Low / Low	Low / High
FINAL_0036	Kulenovic	High / High	High / High	High / High	High / High	High / High	Unclear / Unclear	High / High
FINAL_0038	Li	Low / Low	Low / Low	Low / Low	Unclear / Unclear	Low / Low	Low / Low	Low / Low
FINAL_0040	Berkowitz	Low / Low	Unclear / Unclear	Low / Low	High / High	Unclear / Unclear	Low / Low	High / High
FINAL_0046	Painter	High / High	Unclear / Unclear	Unclear / Unclear	Unclear / Unclear	Unclear / Unclear	Unclear / Unclear	Unclear / Unclear
FINAL_0048	Carpenter	Unclear / Unclear	Unclear / Unclear	Unclear / Unclear	High / High	High / High	Unclear / Unclear	Unclear / Unclear
FINAL_0050	Pandya	High / High	Unclear / Unclear	Unclear / Unclear	High / High	High / High	Unclear / Unclear	High / High
FINAL_0051	Xu	Unclear / Low	Low / Low	Unclear / Unclear	High / High	High / High	Unclear / Unclear	Unclear / Unclear
FINAL_0054	Benaïche	Unclear / High	Unclear / High	Unclear / Low	High / High	High / Unclear	Unclear / Low	High / High
FINAL_0058	Zhang	Low / Low	Unclear / Unclear	Low / Low	Low / Low	Low / Low	Low / Low	Low / Low
FINAL_0059	Gisladottir	Unclear / Unclear	Unclear / Unclear	Low / Low	High / High	Low / Low	Low / Low	Unclear / High
FINAL_0065	Yazdani	Unclear / Unclear	High / High	Low / Low	High / High	Unclear / Unclear	Low / Low	High / High
FINAL_0066	Hosseini	Unclear / Unclear	High / High	Unclear / Unclear	High / High	Unclear / Unclear	Low / Low	Unclear / Unclear
FINAL_0067	Jamil	Low / Low	Low / Low	Low / Low	High / High	Low / Low	Low / Low	Low / Low
FINAL_0070	de Jesus	Unclear / Unclear	Low / Low	Low / Low	High / High	Low / Low	Low / Low	High / High
FINAL_0073	Daluwatte	Low / Low	Low / Low	Low / Low	High / High	High / High	Low / Low	High / Unclear
FINAL_0074	Montastruc	Unclear / Low	Low / Unclear	High / High	High / High	Low / Low	Unclear / Low	High / High
FINAL_0076	Li	Unclear / Unclear	Low / Low	Low / Low	High / High	Low / Low	Low / Low	High / Unclear
FINAL_0082	Hakim	Low / Low	Low / Low	Low / Low	High / High	Low / Low	Low / Low	Low / Low
FINAL_0087	Koon	Unclear / Low	Low / Low	Low / Low	Low / Low	Low / Unclear	Low / Low	Low / Unclear
FINAL_0089	Prioleau	Low / Low	Unclear / Unclear	Low / Low	High / High	Low / Low	Low / Low	High / High
FINAL_0092	Duan	Low / Low	Low / Low	Unclear / High	Low / Low	High / High	Low / Low	High / Unclear
FINAL_0096	Wu	Low / Low	Low / Low	Low / Low	High / High	Low / Low	Low / Low	Low / Low
FINAL_0102	Pariente	Low / High	Low / Low	High / High	High / High	Low / Unclear	Low / High	High / High
FINAL_0105	Wu	Low / Low	Low / Low	Low / Low	High / High	Low / Low	Low / Low	High / High
FINAL_0111	Hu H.	Low / Low	Low / Low	Unclear / Unclear	High / High	Low / Low	Low / Low	Unclear / Unclear
FINAL_0124	Bera A.	Unclear / Unclear	High / High	High / High	High / High	High / High	Low / Low	High / High
FINAL_0127	Jamil S.	Low / Low	Low / Low	Low / Low	High / High	Low / Low	Low / Low	Low / Low
FINAL_0136	Wang N.	Unclear / Unclear	Unclear / Unclear	Low / Low	High / High	High / High	Low / Low	High / High
FINAL_0141	Liu J.	Low / Low	Low / Low	Low / Low	High / High	Low / Low	Low / Low	High / High

Study ID	Author	DQ	TD	MT	ED	CS	OR	EV
FINAL_0147	Ito T.	Unclear / Unclear	Low / Low	High / High	High / High	High / High	Low / Low	High / High
FINAL_0170	Ren X.	Unclear / Unclear	Low / Low	High / High	High / High	Low / Low	Low / Low	Unclear / Unclear
FINAL_0184	Abate A.	Low / Low	Low / Low	Low / Low	High / High	Low / Low	Low / Low	High / High
FINAL_0192	Dou Y.	High / High	High / High	Low / Low	High / High	Low / Low	Unclear / Unclear	High / High
FINAL_0197	Bertalan Á.	High / Low	Low / Low	Unclear / High	Low / Low	Low / Low	Low / Low	Unclear / Low
FINAL_0210	Sridharan K.	Low / Low	Low / Low	Unclear / Unclear	High / High	Low / Low	Low / Low	High / High
FINAL_0211	Osheba A.	High / High	High / High	High / High	High / High	High / High	Unclear / Unclear	High / High
FINAL_0217	Wang Z.	Low / Low	Unclear / Unclear	Unclear / Unclear	High / High	Low / Low	Low / Low	High / High
FINAL_0224	De Vito G.	Low / Low	Low / Low	Low / Low	Low / Low	Unclear / Unclear	Low / Low	Low / Low
FINAL_0226	Li H.	Unclear / Unclear	High / High	Low / Low	High / High	Low / Low	Low / Low	Unclear / Unclear
FINAL_0246	Socrates V.	Low / Low	Low / Low	Low / Low	High / High	Low / Low	Low / Low	High / High
FINAL_0248	Bakouny Z.	High / High	High / High	High / High	High / High	Low / Low	Low / Low	High / High
FINAL_0249	Li Y.	High / Low	Unclear / Low	Low / Low	High / High	Low / Low	Low / Low	High / High
FINAL_0263	Javed M.	Unclear / Unclear	Unclear / Unclear	Unclear / Unclear	High / High	High / High	Unclear / Unclear	Low / Low
FINAL_0271	Sheikh M.S.	High / High	High / High	Unclear / Unclear	High / High	Unclear / Unclear	Unclear / Unclear	High / High
FINAL_0273	Qiu Y.	Low / Unclear	Low / Unclear	Low / Low	Low / High	Low / Low	Low / Low	Unclear / Low
FINAL_0276	Zou H.	Unclear / Unclear	Low / Unclear	Low / Low	Low / Low	Low / Low	Low / Low	Low / Low
FINAL_0277	He C.	Low / Low	Low / Low	Low / Low	Unclear / High	Low / Low	Low / Low	Low / High
FINAL_0278	Guillot J.	Unclear / Unclear	Low / Low	Unclear / Unclear	High / High	Unclear / Unclear	Unclear / Unclear	High / High
FINAL_0298	Bužančić I.	Low / Low	Low / Low	Unclear / Unclear	Unclear / Unclear	Low / Low	Low / Low	Low / Low
FINAL_0304	Yin H.	Low / Low	Unclear / Low	Low / Low	Unclear / Low	Low / Low	Low / Low	Unclear / Unclear
FINAL_0307	Dezoteux F.	Unclear / Unclear	Low / Low	Unclear / Unclear	High / High	Low / Low	Low / Low	High / High
FINAL_0311	Zheng Y.	Low / Low	Low / Low	Low / Low	Unclear / High	Low / Low	Low / Low	Low / High
FINAL_0318	Gitman V.	Low / Low	Low / Low	Low / Low	Unclear / Unclear	Low / Low	Low / Low	Low / Low
FINAL_0332	Kim M.	Low / Low	Low / Low	Low / Low	Low / Low	Low / Low	Low / Low	Low / Low
FINAL_0337	Prioleau H.	Low / Low	Low / Low	Low / High	High / High	Low / Low	Low / Low	Low / High
FINAL_0345	Prioleau H.	Low / Low	Low / High	Low / Low	High / High	Low / High	Low / Low	Low / High
FINAL_0353	Elbiach O.	Low / Low	Low / Low	Low / Low	Low / High	Low / Low	Low / Low	Low / High
FINAL_0386	Burnette H.	Low / Low	Low / Low	Unclear / Unclear	Low / Low	Low / Low	Low / Low	Low / Low
FINAL_0392	Painter J.L.	Low / Low	Low / Low	Low / Low	High / High	Low / Low	Low / Low	Low / Low
FINAL_0394	Sun V.H.	Low / Low	Low / Low	Low / Low	Low / Low	Low / Low	Low / Low	Low / Low
FINAL_0399	Wakabayashi S.	High / High	Unclear / Unclear	Unclear / Unclear	High / High	High / High	High / High	High / High
FINAL_0407	Ying L.	Low / Low	Low / Low	Low / Low	High / High	Low / Low	Low / Low	High / High
FINAL_0416	Khademi S.	Low / Low	Low / Low	Low / Low	High / Unclear	Low / High	Low / Low	High / High
FINAL_0435	Vismaya S.	Unclear / Unclear	Low / Low	Low / Low	Unclear / Unclear	Low / Low	Low / Low	Unclear / Unclear
FINAL_0439	Hu H.	Low / Low	Low / Low	Low / Low	Low / Low	Low / Low	Low / Low	Low / Low
FINAL_0460	Alshehri B.M.J.	High / High	High / High	Low / Low	High / High	High / High	Low / Low	High / High
FINAL_0461	Chandra M.	Unclear / Low	Low / Low	Low / Low	High / High	Low / Low	Low / Low	High / High
FINAL_0468	Fu W.	High / High	Unclear / Unclear	Low / Low	High / High	Unclear / Unclear	Unclear / Unclear	High / High
FINAL_0475	Zhang R.	Low / Unclear	Low / Unclear	Low / Low	Low / Low	Low / Low	Low / Low	Low / Low
FINAL_0476	Elbiach O.	Low / Low	Low / Low	Low / Low	Low / High	Low / Low	Low / Low	Low / Unclear
FINAL_0479	Liu Q.	Low / Low	Low / Low	Unclear / Unclear	High / High	Low / Low	Low / Low	High / High

Study ID	Author	DQ	TD	MT	ED	CS	OR	EV
FINAL_0486	Kim J.	High / Low	High / Unclear	Low / Low	High / High	Low / Low	Low / Low	High / High
FINAL_0494	Wei	Unclear / Unclear	High / High	High / High	High / High	High / High	Low / Low	High / Unclear
FINAL_0505	Dietrich	Low / Low	Low / Low	Low / Low	High / High	Low / Low	Low / Low	Unclear / Unclear

Table S8. Inter-rater agreement for signalling questions and domain-level judgements.

Level	Metric	Paired n	Matches	Agreement	Cohen's κ	95% CI	Interpretation
Question	DQ_Q1	83	79	95.2%	0.697	0.321–0.936	Substantial
Question	DQ_Q2	83	75	90.4%	0.779	0.621–0.908	Substantial
Question	DQ_Q3	83	78	94.0%	0.835	0.670–0.962	Almost perfect
Question	TD_Q1	83	81	97.6%	0.846	0.556–1.000	Almost perfect
Question	TD_Q2	83	74	89.2%	0.774	0.621–0.900	Substantial
Question	MT_Q1	83	79	95.2%	0.803	0.588–0.956	Almost perfect
Question	MT_Q2	83	80	96.4%	0.929	0.841–1.000	Almost perfect
Question	ED_Q1	83	81	97.6%	0.878	0.650–1.000	Almost perfect
Question	ED_Q2	83	75	90.4%	0.773	0.612–0.907	Substantial
Question	CS_Q1	83	80	96.4%	0.913	0.796–1.000	Almost perfect
Question	CS_Q2	83	78	94.0%	0.863	0.732–0.970	Almost perfect
Question	OR_Q1	83	80	96.4%	0.851	0.642–1.000	Almost perfect
Question	OR_Q2	83	82	98.8%	0.941	0.781–1.000	Almost perfect
Question	EV_Q1	83	75	90.4%	0.827	0.705–0.933	Almost perfect
Question	EV_Q2	83	71	85.5%	0.761	0.632–0.877	Substantial
Domain	Data Quality	83	72	86.7%	0.765	0.624–0.884	Substantial
Domain	Task Definition	83	73	88.0%	0.776	0.635–0.894	Substantial
Domain	Model Transparency	83	79	95.2%	0.908	0.811–0.978	Almost perfect
Domain	Evaluation Design	83	75	90.4%	0.775	0.614–0.907	Substantial
Domain	Comparator Strength	83	78	94.0%	0.880	0.766–0.974	Almost perfect
Domain	Outcome Reporting	83	80	96.4%	0.870	0.692–1.000	Almost perfect
Domain	External Validity	83	68	81.9%	0.703	0.562–0.829	Substantial
Question	All signalling questions (pooled)	1,245	1,168	93.8%	0.863	0.809–0.911	Almost perfect
Domain	All domains (pooled)	581	525	90.4%	0.837	0.772–0.895	Almost perfect

Cohen's κ is unweighted for nominal categories. Confidence intervals are 95% percentile cluster-bootstrap intervals based on 200,000 study-level resamples, thereby preserving the clustering of multiple judgements within each study. Degenerate resamples yielding an undefined κ were omitted for individual metrics before percentile estimation (minimum valid resamples, 199,962); all 200,000 resamples were valid for the pooled estimates.

Table S9. Complete database-specific search strategies, coverage period, and final search date.

Database	Final search date	Coverage	Full search strategy
PubMed	30 March 2026	1 January 2022–30 March 2026	(("large language model"[Title/Abstract] OR "large language models"[Title/Abstract] OR LLM[Title/Abstract] OR GPT[Title/Abstract] OR ChatGPT[Title/Abstract] OR Gemini[Title/Abstract] OR Claude[Title/Abstract] OR Llama[Title/Abstract]) AND ("adverse drug reaction"[Title/Abstract] OR "adverse drug reactions"[Title/Abstract] OR ADR[Title/Abstract] OR "adverse drug event"[Title/Abstract] OR "adverse drug events"[Title/Abstract] OR ADE[Title/Abstract] OR pharmacovigilance[Title/Abstract] OR "drug safety"[Title/Abstract] OR "medication safety"[Title/Abstract]))
Scopus	30 March 2026	1 January 2022–30 March 2026	TITLE-ABS-KEY(("large language model*" OR LLM OR GPT OR ChatGPT OR Gemini OR Claude OR Llama) AND ("adverse drug reaction*" OR ADR OR "adverse drug event*" OR ADE OR pharmacovigilance OR "drug safety" OR "medication safety"))
Web of Science	30 March 2026	1 January 2022–30 March 2026	TS=(("large language model*" OR LLM OR GPT OR ChatGPT OR Gemini OR Claude OR Llama) AND ("adverse drug reaction*" OR ADR OR "adverse drug event*" OR ADE OR pharmacovigilance OR "drug safety" OR "medication safety"))

Table S10. Paired pre-consensus signalling-question-level judgements for both independent reviewers across all 83 included studies.
Each cell reports Rater 1 / Rater 2. Y = Yes; N = No; U = Unclear. Question codes and full wording are defined in Supplementary Table S6.

Study ID	Author	DQ1	DQ2	DQ3	TD1	TD2	MT1	MT2	ED1	ED2	CS1	CS2	OR1	OR2	EV1	EV2
FINAL_0001	Huang	Y / Y	Y / Y	Y / Y	Y / Y	U / U	Y / Y	U / U	Y / Y	N / N	Y / Y	Y / Y	Y / Y	Y / Y	N / N	U / U
FINAL_0004	Kopacheva	Y / Y	U / U	N / N	N / N	U / U	Y / Y	N / N	U / U	N / N	N / N	N / N	Y / Y	Y / Y	N / N	N / N
FINAL_0007	Sicard	Y / Y	Y / Y	Y / Y	Y / Y	Y / Y	Y / Y	Y / Y	Y / Y	N / N	Y / Y	N / N	Y / Y	Y / Y	N / N	U / U
FINAL_0008	Ong	Y / Y	Y / Y	Y / Y	Y / Y	Y / Y	Y / Y	Y / Y	Y / Y	N / N	Y / Y	Y / Y	Y / Y	Y / Y	Y / Y	U / U
FINAL_0018	Pais	Y / Y	Y / Y	Y / Y	Y / Y	Y / Y	Y / Y	Y / Y	Y / Y	Y / Y	Y / Y	Y / Y	Y / Y	Y / Y	Y / Y	Y / Y
FINAL_0020	Blotske	Y / Y	Y / Y	Y / Y	Y / Y	Y / Y	Y / Y	Y / Y	Y / Y	N / N	U / U	U / U	Y / Y	Y / Y	N / N	U / U
FINAL_0023	Deng	Y / Y	Y / Y	Y / Y	Y / Y	Y / Y	Y / Y	Y / Y	Y / Y	N / N	Y / Y	Y / Y	Y / Y	Y / Y	N / N	U / U
FINAL_0029	Li	Y / Y	Y / Y	Y / Y	Y / Y	Y / Y	Y / Y	Y / Y	Y / Y	Y / Y	Y / Y	Y / Y	Y / Y	Y / Y	Y / Y	Y / Y
FINAL_0033	Ma	Y / Y	Y / Y	Y / Y	Y / Y	Y / U	Y / Y	Y / Y	Y / Y	Y / N	Y / Y	Y / Y	Y / Y	Y / Y	Y / N	Y / U
FINAL_0036	Kulenovic	U / Y	U / N	N / N	Y / Y	N / N	N / U	N / N	N / Y	N / N	N / Y	N / N	U / U	Y / Y	N / N	N / N
FINAL_0038	Li	Y / Y	Y / Y	Y / Y	Y / Y	Y / Y	Y / Y	Y / Y	Y / Y	U / U	Y / Y	Y / Y	Y / Y	Y / Y	Y / Y	Y / Y
FINAL_0040	Berkowitz	Y / Y	Y / Y	Y / Y	Y / Y	U / U	Y / Y	Y / Y	Y / Y	N / N	U / U	Y / Y	Y / Y	Y / Y	N / N	U / U
FINAL_0046	Painter	Y / Y	N / N	U / U	U / U	Y / Y	U / U	U / U	U / U	Y / Y	U / U	U / U	U / U	U / U	U / U	U / U
FINAL_0048	Carpenter	Y / Y	Y / Y	U / U	U / U	Y / Y	U / U	U / U	Y / Y	N / N	N / N	U / U	U / U	U / U	U / U	U / U
FINAL_0050	Pandya	Y / Y	N / N	U / U	U / U	Y / Y	U / U	U / U	U / U	N / N	N / N	U / U	U / U	U / U	N / N	N / N
FINAL_0051	Xu	Y / Y	Y / Y	U / Y	Y / Y	Y / Y	U / U	U / U	Y / Y	N / N	N / N	U / N	U / Y	U / U	U / U	U / U
FINAL_0054	Benaïche	Y / Y	U / N	Y / Y	U / U	Y / N	U / Y	Y / Y	U / U	N / N	N / Y	Y / U	Y / Y	U / Y	N / N	U / U
FINAL_0058	Zhang	Y / Y	Y / Y	Y / Y	Y / Y	U / U	Y / Y	Y / Y	Y / Y	Y / Y	Y / Y	Y / Y	Y / Y	Y / Y	Y / Y	Y / Y
FINAL_0059	Gisladottir	Y / Y	Y / Y	U / U	Y / Y	U / U	Y / Y	Y / Y	Y / Y	N / N	Y / Y	Y / Y	Y / Y	Y / Y	U / N	Y / Y
FINAL_0065	Yazdani	Y / Y	U / U	Y / Y	Y / Y	N / N	Y / Y	Y / Y	Y / Y	N / N	U / U	U / U	Y / Y	Y / Y	N / N	N / N
FINAL_0066	Hosseini	Y / Y	U / U	Y / Y	Y / Y	N / N	Y / Y	U / U	Y / Y	N / N	U / U	Y / Y	Y / Y	Y / Y	Y / Y	U / U
FINAL_0067	Jamil	Y / Y	Y / Y	Y / Y	Y / Y	Y / Y	Y / Y	Y / Y	Y / Y	N / N	Y / Y	Y / Y	Y / Y	Y / Y	Y / Y	Y / Y
FINAL_0070	de Jesus	U / U	Y / Y	Y / Y	Y / Y	Y / Y	Y / Y	Y / Y	Y / Y	N / N	Y / Y	Y / Y	Y / Y	Y / Y	N / N	U / U
FINAL_0073	Daluwatte	Y / Y	Y / Y	Y / Y	Y / Y	Y / Y	Y / Y	Y / Y	Y / Y	N / N	N / N	N / N	Y / Y	Y / Y	Y / Y	N / U
FINAL_0074	Montastruc	Y / Y	Y / Y	U / Y	Y / U	Y / Y	Y / N	N / N	Y / Y	N / N	Y / Y	Y / Y	U / Y	Y / Y	N / N	N / U
FINAL_0076	Li	Y / Y	Y / Y	U / U	Y / Y	Y / Y	Y / Y	Y / Y	Y / Y	N / N	Y / Y	Y / Y	Y / Y	Y / Y	Y / Y	N / U
FINAL_0082	Hakim	Y / Y	Y / Y	Y / Y	Y / Y	Y / Y	Y / Y	Y / Y	Y / Y	N / N	Y / Y	Y / Y	Y / Y	Y / Y	Y / Y	Y / Y
FINAL_0087	Koon	Y / Y	U / Y	Y / Y	Y / Y	Y / Y	Y / Y	Y / Y	Y / Y	Y / Y	Y / Y	Y / U	Y / Y	Y / Y	Y / Y	Y / U
FINAL_0089	Prioleau	Y / Y	Y / Y	Y / Y	Y / Y	U / U	Y / Y	Y / Y	Y / Y	N / N	Y / Y	Y / Y	Y / Y	Y / Y	N / N	U / U
FINAL_0092	Duan	Y / Y	Y / Y	Y / Y	Y / Y	Y / Y	Y / Y	U / N	Y / Y	Y / Y	N / N	N / N	Y / Y	Y / Y	Y / Y	N / U
FINAL_0096	Wu	Y / Y	Y / Y	Y / Y	Y / Y	Y / Y	Y / Y	Y / Y	Y / Y	N / N	Y / Y	Y / Y	Y / Y	Y / Y	Y / Y	Y / Y
FINAL_0102	Pariente	Y / Y	Y / Y	Y / N	Y / Y	Y / Y	Y / Y	N / N	Y / Y	N / N	Y / Y	Y / U	Y / N	Y / Y	N / N	N / U
FINAL_0105	Wu	Y / Y	Y / Y	Y / Y	Y / Y	Y / Y	Y / Y	Y / Y	Y / Y	N / N	Y / Y	Y / Y	Y / Y	Y / Y	N / N	U / U
FINAL_0111	Hu H.	Y / Y	Y / Y	Y / Y	Y / Y	Y / Y	Y / Y	U / U	Y / Y	N / N	Y / Y	Y / Y	Y / Y	Y / Y	Y / Y	U / U
FINAL_0124	Bera A.	Y / Y	Y / Y	U / U	Y / Y	N / N	Y / Y	N / N	Y / Y	N / N	N / N	Y / Y	Y / Y	Y / Y	N / N	N / U
FINAL_0127	Jamil S.	Y / Y	Y / Y	Y / Y	Y / Y	Y / Y	Y / Y	Y / Y	Y / Y	N / N	Y / Y	Y / Y	Y / Y	Y / Y	Y / Y	Y / Y
FINAL_0136	Wang N.	U / U	Y / Y	Y / Y	Y / Y	U / U	Y / Y	Y / Y	Y / Y	N / N	N / N	U / U	Y / Y	Y / Y	N / N	U / U
FINAL_0141	Liu J.	Y / Y	Y / Y	Y / Y	Y / Y	Y / Y	Y / Y	Y / Y	Y / Y	N / N	Y / Y	Y / Y	Y / Y	Y / Y	N / N	U / U
FINAL_0147	Ito T.	Y / Y	U / U	Y / Y	Y / Y	Y / Y	Y / Y	N / N	Y / Y	N / N	U / U	N / N	Y / Y	Y / Y	N / N	N / N
FINAL_0170	Ren X.	Y / Y	Y / Y	U / U	Y / Y	Y / Y	Y / Y	N / N	Y / Y	N / N	Y / Y	Y / Y	Y / Y	Y / Y	Y / Y	U / U
FINAL_0184	Abate A.	Y / Y	Y / Y	Y / Y	Y / Y	Y / Y	Y / Y	Y / Y	Y / Y	N / N	Y / Y	Y / Y	Y / Y	Y / Y	N / N	Y / Y
FINAL_0192	Dou Y.	Y / Y	N / N	Y / Y	Y / Y	N / N	Y / Y	Y / Y	U / U	N / N	Y / Y	Y / Y	Y / Y	U / U	N / N	U / U
FINAL_0197	Bertalan Á.	Y / Y	N / Y	Y / Y	Y / Y	Y / Y	U / Y	U / N	Y / Y	Y / Y	Y / Y	Y / Y	Y / Y	Y / Y	Y / Y	U / Y
FINAL_0210	Sridharan K.	Y / Y	Y / Y	Y / Y	Y / Y	Y / Y	U / U	U / U	Y / Y	N / N	Y / Y	Y / Y	Y / Y	Y / Y	N / N	U / U
FINAL_0211	Osheba A.	Y / Y	N / N	U / U	Y / Y	N / N	Y / Y	N / N	Y / Y	N / N	N / N	N / N	U / U	Y / Y	N / N	U / U
FINAL_0217	Wang Z.	Y / Y	Y / Y	Y / Y	Y / Y	U / U	Y / Y	U / U	Y / Y	N / N	Y / Y	Y / Y	Y / Y	Y / Y	N / N	U / U
FINAL_0224	De Vito G.	Y / Y	Y / Y	Y / Y	Y / Y	Y / Y	Y / Y	Y / Y	Y / Y	Y / Y	Y / Y	U / U	Y / Y	Y / Y	Y / Y	Y / Y
FINAL_0226	Li H.	Y / Y	U / U	Y / Y	Y / Y	N / N	Y / Y	Y / Y	Y / Y	N / N	Y / Y	Y / Y	Y / Y	Y / Y	Y / Y	U / U
FINAL_0246	Socrates V.	Y / Y	Y / Y	Y / Y	Y / Y	Y / Y	Y / Y	Y / Y	Y / Y	N / N	Y / Y	Y / Y	Y / Y	Y / Y	N / N	U / U
FINAL_0248	Bakouny Z.	Y / Y	U / U	N / N	Y / Y	N / N	Y / Y	N / N	Y / Y	N / N	Y / Y	Y / Y	Y / Y	Y / Y	N / N	U / U
FINAL_0249	Li Y.	U / Y	N / Y	Y / Y	Y / Y	U / Y	Y / Y	Y / Y	U / Y	N / N	Y / Y	Y / Y	Y / Y	Y / Y	N / N	U / U
FINAL_0263	Javed M.	Y / Y	U / U	Y / Y	Y / Y	U / U	Y / Y	U / U	Y / Y	N / N	N / N	N / N	U / U	U / U	Y / Y	Y / Y
FINAL_0271	Sheikh M.S.	N / N	N / N	N / N	Y / Y	N / N	Y / Y	U / U	N / N	N / N	U / U	U / U	U / U	U / U	N / N	U / U

Study ID	Author	DQ1	DQ2	DQ3	TD1	TD2	MT1	MT2	ED1	ED2	CS1	CS2	OR1	OR2	EV1	EV2
FINAL_0273	Qiu Y.	Y / Y	Y / U	Y / Y	Y / Y	Y / U	Y / Y	Y / Y	Y / Y	Y / N	Y / Y	Y / Y	Y / Y	Y / Y	Y / Y	U / Y
FINAL_0276	Zou H.	Y / Y	U / U	Y / Y	Y / Y	Y / U	Y / Y	Y / Y	Y / Y	Y / Y	Y / Y	Y / Y	Y / Y	Y / Y	Y / Y	Y / Y
FINAL_0277	He C.	Y / Y	Y / Y	Y / Y	Y / Y	Y / Y	Y / Y	Y / Y	Y / Y	U / N	Y / Y	Y / Y	Y / Y	Y / Y	Y / N	Y / Y
FINAL_0278	Guillot J.	Y / Y	U / U	U / U	Y / Y	Y / Y	U / U	U / U	Y / Y	N / N	Y / Y	U / U	U / U	Y / Y	N / N	Y / Y
FINAL_0298	Bužančić I.	Y / Y	Y / Y	Y / Y	Y / Y	Y / Y	Y / Y	U / U	Y / Y	U / U	Y / Y	Y / Y	Y / Y	Y / Y	Y / Y	Y / Y
FINAL_0304	Yin H.	Y / Y	Y / Y	Y / Y	Y / Y	U / Y	Y / Y	Y / Y	Y / Y	U / Y	Y / Y	Y / Y	Y / Y	Y / Y	Y / Y	U / U
FINAL_0307	Dezoteux F.	Y / Y	Y / Y	U / U	Y / Y	Y / Y	Y / Y	U / U	Y / Y	N / N	Y / Y	Y / Y	Y / Y	Y / Y	N / N	Y / Y
FINAL_0311	Zheng Y.	Y / Y	Y / Y	Y / Y	Y / Y	Y / Y	Y / Y	Y / Y	Y / Y	U / N	Y / Y	Y / Y	Y / Y	Y / Y	Y / N	Y / Y
FINAL_0318	Gitman V.	Y / Y	Y / Y	Y / Y	Y / Y	Y / Y	Y / Y	Y / Y	Y / Y	U / U	Y / Y	Y / Y	Y / Y	Y / Y	Y / Y	Y / Y
FINAL_0332	Kim M.	Y / Y	Y / Y	Y / Y	Y / Y	Y / Y	Y / Y	Y / Y	Y / Y	Y / Y	Y / Y	Y / Y	Y / Y	Y / Y	Y / Y	Y / Y
FINAL_0337	Prioleau H.	Y / Y	Y / Y	Y / Y	Y / Y	Y / Y	Y / Y	Y / N	Y / Y	N / N	Y / Y	Y / Y	Y / Y	Y / Y	Y / N	Y / Y
FINAL_0345	Prioleau H.	Y / Y	Y / Y	Y / Y	Y / Y	Y / N	Y / Y	Y / Y	Y / Y	N / N	Y / N	Y / Y	Y / Y	Y / Y	Y / N	Y / Y
FINAL_0353	Elbiach O.	Y / Y	Y / Y	Y / Y	Y / Y	Y / Y	Y / Y	Y / Y	Y / Y	Y / N	Y / Y	Y / Y	Y / Y	Y / Y	Y / N	Y / Y
FINAL_0386	Burnette H.	Y / Y	Y / Y	Y / Y	Y / Y	Y / Y	Y / Y	U / U	Y / Y	Y / Y	Y / Y	Y / Y	Y / Y	Y / Y	Y / Y	Y / Y
FINAL_0392	Painter J.L.	Y / Y	Y / Y	Y / Y	Y / Y	Y / Y	Y / Y	Y / Y	Y / Y	N / N	Y / Y	Y / Y	Y / Y	Y / Y	Y / Y	Y / Y
FINAL_0394	Sun V.H.	Y / Y	Y / Y	Y / Y	Y / Y	Y / Y	Y / Y	Y / Y	Y / Y	Y / Y	Y / Y	Y / Y	Y / Y	Y / Y	Y / Y	Y / Y
FINAL_0399	Wakabayashi S.	Y / Y	N / N	U / U	U / U	Y / Y	U / U	Y / Y	N / N	N / N	N / N	N / N	N / N	U / U	N / N	U / U
FINAL_0407	Ying L.	Y / Y	Y / Y	Y / Y	Y / Y	Y / Y	Y / Y	Y / Y	Y / Y	N / N	Y / Y	Y / Y	Y / Y	Y / Y	N / N	Y / Y
FINAL_0416	Khademi S.	Y / Y	Y / Y	Y / Y	Y / Y	Y / Y	Y / Y	Y / Y	Y / Y	N / U	Y / Y	Y / N	Y / Y	Y / Y	N / N	Y / Y
FINAL_0435	Vismaya S.	Y / Y	U / U	Y / Y	Y / Y	Y / Y	Y / Y	Y / Y	Y / Y	U / U	Y / Y	Y / Y	Y / Y	Y / Y	U / U	Y / Y
FINAL_0439	Hu H.	Y / Y	Y / Y	Y / Y	Y / Y	Y / Y	Y / Y	Y / Y	Y / Y	Y / Y	Y / Y	Y / Y	Y / Y	Y / Y	Y / Y	Y / Y
FINAL_0460	Alshehri B.M.J.	N / N	U / U	Y / Y	Y / Y	N / N	Y / Y	Y / Y	U / U	N / N	N / N	Y / Y	Y / Y	Y / Y	N / N	N / N
FINAL_0461	Chandra M.	U / Y	Y / Y	Y / Y	Y / Y	Y / Y	Y / Y	Y / Y	Y / Y	N / N	Y / Y	Y / Y	Y / Y	Y / Y	N / N	U / U
FINAL_0468	Fu W.	N / N	U / U	U / U	Y / Y	U / U	Y / Y	Y / Y	Y / Y	N / N	Y / Y	U / U	U / U	U / U	Y / Y	N / N
FINAL_0475	Zhang R.	Y / Y	Y / U	Y / Y	Y / Y	Y / U	Y / Y	Y / Y	Y / Y	Y / Y	Y / Y	Y / Y	Y / Y	Y / Y	Y / Y	Y / Y
FINAL_0476	Elbiach O.	Y / Y	Y / Y	Y / Y	Y / Y	Y / Y	Y / Y	Y / Y	Y / Y	Y / N	Y / Y	Y / Y	Y / Y	Y / Y	Y / Y	Y / U
FINAL_0479	Liu Q.	Y / Y	Y / Y	Y / Y	Y / Y	Y / Y	U / U	U / U	Y / Y	N / N	Y / Y	Y / Y	Y / Y	Y / Y	N / N	U / U
FINAL_0486	Kim J.	N / Y	Y / Y	U / Y	Y / Y	N / U	Y / Y	Y / Y	Y / Y	N / N	Y / Y	Y / Y	Y / Y	Y / Y	N / N	N / U
FINAL_0494	Wei	Y / Y	Y / U	U / Y	N / Y	N / N	N / N	N / N	Y / Y	N / N	N / N	N / N	Y / Y	Y / Y	N / U	U / U
FINAL_0505	Dietrich	Y / Y	Y / Y	Y / Y	Y / Y	Y / Y	Y / Y	Y / Y	Y / Y	N / N	Y / Y	Y / Y	Y / Y	Y / Y	Y / Y	U / U
